# Nanohydroxyapatite composites' toxicity and biosafety for safe clinical translation

**DOI:** 10.1039/d6na00491a

**Published:** 2026-08-03

**Authors:** Shikha Awasthi

**Affiliations:** a Jagran School of Pharmacy, Jagran Lakecity University Bhopal - 462044 Madhya Pradesh India awas.shikha2212@gmail.com

## Abstract

Nanohydroxyapatite (nHAp) is a promising biomaterial due to its structural similarity with the mineral phase of natural bone, high bioactivity, and broad applications in biomedical fields. Although this field is growing rapidly, there is no dedicated review which systematically offers an overview on toxicity and bio-safety profile of nHAp composites. The researchers reported that till 50–100 µg mL^−1^ concentration of nHAp, cell viability was 80–90%, but as the concentration reached around 100–200 µg mL^−1^, it caused oxidative stress, inflammation, decreased cell proliferation and mitochondrial dysfunction. This review aims to give a comparative critical summary of the toxicity aspects of nanohydroxyapatite composites, focusing on cytotoxicity, oxidative stress, genotoxicity and immunological responses. A particular focus is given to dose-dependent behaviour, ion-doping effects, and the effect of the various composite matrices on cellular responses. In addition, the potential toxicity differences between biomedical applications like bone regeneration, drug delivery systems and implant coatings are discussed. The review emphasizes the importance of *in vivo* studies, long-term toxicity testing, and standardized testing protocols for the safe clinical application of nHAp-based composites. In summary, nanohydroxyapatite composites hold great promise for advanced biomedical applications; nonetheless, their toxicity profile is complex and context-dependent, necessitating a sophisticated comprehension of their safety issues and therapeutic advantages.

## Abstract 2

nHAp composites are promising, but these composites can trigger a number of toxicity-related responses depending on the particle characteristics, composite composition, dosage, and biological exposure conditions (the figure was created using BioRender).

## Graphical abstract

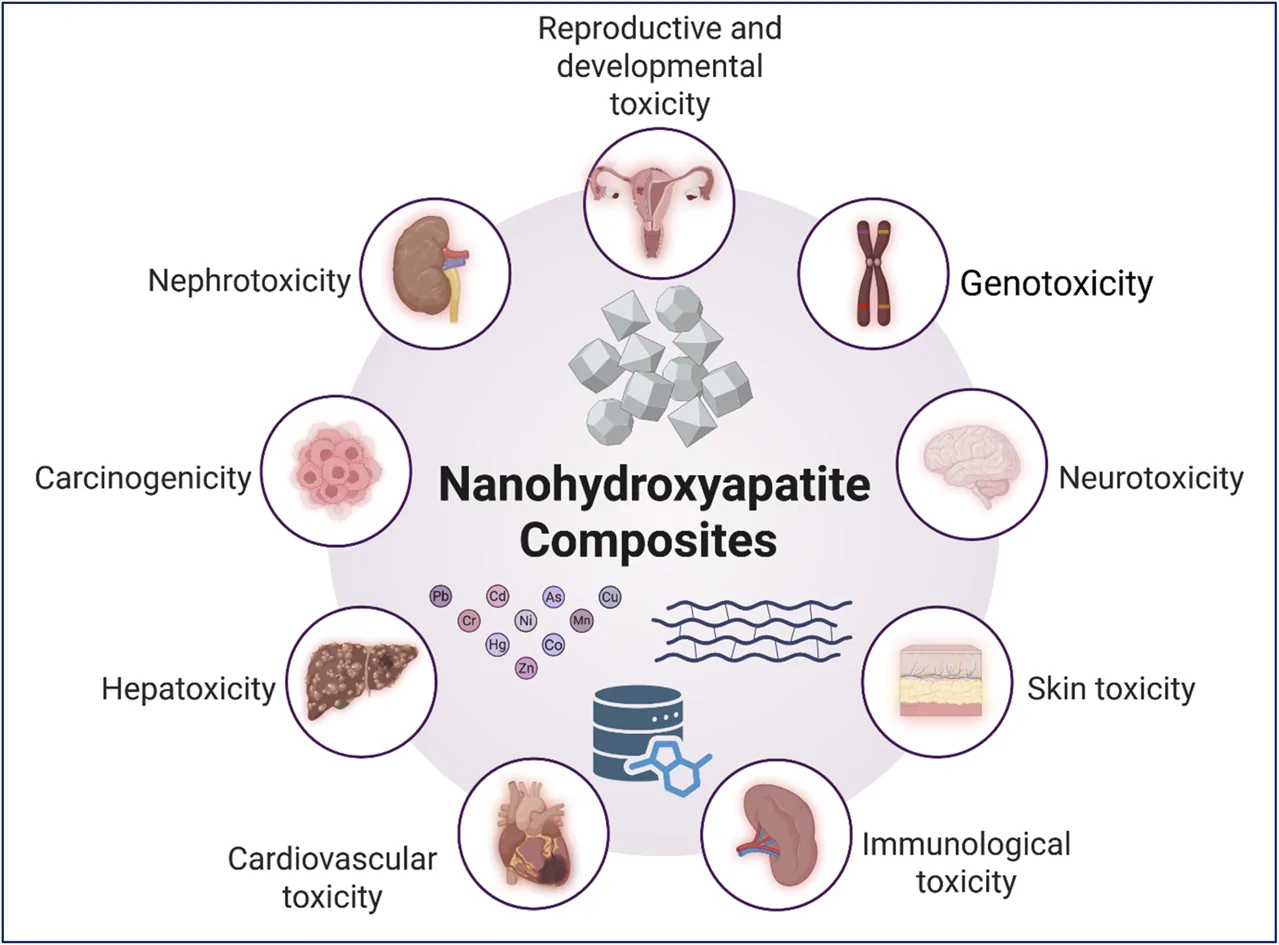

## 1. Introduction

Hydroxyapatite (HAp) is the major component (inorganic) of human bone and teeth (∼60–70%).^1–3^ Due to its excellent biocompatibility, osteoconductivity, and chemical similarity to natural hard tissues, HAp has been extensively explored in various clinical applications, such as bone tissue engineering, dental materials, implant coatings, and drug delivery systems.^4–6^ The pure hydroxyapatite, however, is very brittle and has poor mechanical properties, which limit its direct load-bearing applications.^7^ In order to address these limitations, considerable research efforts have been focused on the development of nanoscale hydroxyapatite (nHAp) and composite systems of nHAp with polymers, metals, ceramics and carbon-based nanomaterials.^8,9^ Nanohydroxyapatite typically exhibits a particle size in the range of 20–80 nm, with a specific surface area of approximately 50–150 m^2^ g^−1^, depending on synthesis route and morphology.^10^ In comparison to bulk hydroxyapatite (micron-sized, ∼1–10 µm, surface area <10 m^2^ g^−1^), this nanoscale reduction results in significantly enhanced bioactivity, increased protein adsorption, improved osteoblast adhesion, and faster resorption behaviour due to higher surface reactivity and greater ion exchange capacity at the biological interface.^11–13^ The nanoscale features enable the more natural apatite crystals to be reproduced, which leads to faster bone regeneration and better integration with host tissues. As a result, nHAp has become an important material for use in advanced strategies for regenerative medicine and nanomedicine.

Even with these benefits, there are new biological issues when moving from micro-to nanoscale. The surface energy of nHAp is high, its reactivity is high, and it can be internalized by cells, so the safety of nHAp is a concern, especially for long-term or high-dose exposure.^14,15^ Although hydroxyapatite is non-toxic and bio-safe, recent studies indicate that its nano-sized version can have context-dependent biological responses, particularly when used in composite systems.^14^ In recent years, nanohydroxyapatite-based composites have attracted great interest because they can synergistically combine mechanical strength, biological activity, and functional versatility. These composites are generally produced by combining nHAp with natural or synthetic polymers like chitosan, gelatin, collagen, polyvinyl alcohol (PVA), polylactic acid (PLA) or inorganic phases like titanium, zirconia, bioactive glass, and silica-based ceramics.^16,17^ Besides, carbon-based nanomaterials like graphene and carbon nanotubes have also been introduced to improve mechanical reinforcement and electrical conductivity for specific biomedical applications.^18,19^

These composite systems have multifunctional properties, but the presence of secondary phases and interactions at the nanoscale can have a strong effect on the biological response. Therefore, the toxicity of nHAp composites is not only related to the hydroxyapatite itself but also to composite architecture, surface chemistry, ion release behaviour, degradation products, and interaction with biological systems. These factors all influence cellular processes like adhesion, proliferation, differentiation, oxidative stress, inflammatory signalling and apoptosis.^20–22^ One of the most important concerns with nanomaterials, such as nHAp, is their capacity to cause oxidative stress by the creation of reactive oxygen species (ROS). Overproduction of ROS may affect the redox balance in cells, cause damage to proteins, lipids, and DNA, and result in dysfunction or death of the cells.^22,23^ Some studies have also shown that nHAp can induce mild to moderate oxidative stress response under certain conditions, including high concentration, long exposure time, or surface modification. These effects can be reduced or enhanced in composite systems depending on the type of secondary components.^24^

Shape-dependent toxicity has been reported, and needle-shaped or rod-like particles are more biologically reactive than spherical particles, suggesting that shape is an important factor in nanosafety evaluation.^20^ The toxicity of nHAp composites is further complicated by the addition of ion doping and surface functionalization. Biologically active ions like silver (Ag^+^), copper (Cu^2+^), zinc (Zn^2+^), strontium (Sr^2+^), and fluoride (F^−^) are commonly incorporated to improve antimicrobial properties and osteogenic potential.^21^ But, if there is too much release of ions, it can lead to cytotoxic effects depending on the concentration and the length of exposure. Thus, the use of metallic or carbon-based reinforcements can enhance mechanical properties, but may also create new biological hazards from the toxicity or degradation of the reinforcement itself.^25,26^

Apart from cytotoxicity, immunological responses are now known to be an important factor in the safety evaluation of nanomaterials. Nanohydroxyapatite composites can modulate the function of immune cells, including cytokine production, inflammatory pathways, and phagocytosis, by interacting with macrophages. These materials can either induce pro-inflammatory or anti-inflammatory and regenerative phenotypes, depending on their physicochemical properties. Thus, immunotoxicity testing is crucial to the understanding of the long-term biocompatibility of nHAp-based systems.^23^ Another key but less studied area in the case of nHAp composites is genotoxicity.^15,27^ In some studies, potential DNA damage, chromosomal aberrations and mitochondrial dysfunction have been reported, especially with high dose exposure or specific nanostructured forms. Most of the literature data available, however, are short-term, and it is difficult to make definite conclusions on genetic safety.

Although there are many studies on nanohydroxyapatite-based materials, most of them are focused on the synthesis, functionalization, and application potential of these materials, and not on detailed toxicity profiling.^17,28,29^ Most *in vitro* studies are short-term assays (MTT or live/dead staining) that offer only a limited view of chronic effects, systemic toxicity and *in vivo* behaviour. Moreover, there are no standardised protocols for assessing nanosafety, which results in discrepancies in the reported results. One of the major challenges is the translation of *in vitro* results to *in vivo* systems. The toxicity of nanomaterials is greatly affected by their biological complexity, such as protein corona formation, biodistribution, metabolic degradation, and clearance mechanisms. Thus, the physiological response that is obtained in simplified laboratory conditions may not be fully representative of that which occurs *in vivo*.

With the growing biomedical applications of nanohydroxyapatite composites, it is crucial to have a thorough understanding of their toxicity profile for safe clinical translation.^24^ This review aims to critically assess the current literature on the toxicity of nanohydroxyapatite composites, focusing on the main parameters that affect the biological responses of these materials, such as the physicochemical properties of the nanoparticles, the composition of the composites, their dose dependence, surface modifications, and complex interactions between the bio and nano components. Moreover, toxicity behaviour in various biomedical applications is addressed, giving an integrated and application-oriented view of their safety profile. Importantly, although nanohydroxyapatite-based systems are considered biocompatible and have been extensively studied for their use in regenerative and therapeutic applications, the available toxicity data are highly fragmented, inconsistent, and mostly short-term *in vitro.* There is still no systematic collection of these isolated results, particularly from the point of view of composite-dependent behaviour, in the current literature. This review thus addresses a critical knowledge gap by collating the scattered evidence, pointing to conflicting reports, and identifying under-researched toxicity pathways, including immunological responses, oxidative stress pathways, and long-term *in vivo* effects. In addition, it highlights the lack of standardization of nanosafety evaluation protocols, which hinders the ability to make reliable comparisons across studies. To conclude, nanohydroxyapatite composites have a great potential in the field of regenerative medicine and biomedical engineering, but their toxicity profile is complex, context-dependent and not sufficiently standardized. This review is timely and critical, not only summarizing existing knowledge but also identifying key research gaps and future directions needed for the rational design of safe, clinically translatable nanohydroxyapatite-based systems. Thus, there is a need for a balanced and evidence-based evaluation of their therapeutic potential and possible risks for their safe progression in biomedical applications.

**Fig. 1:**
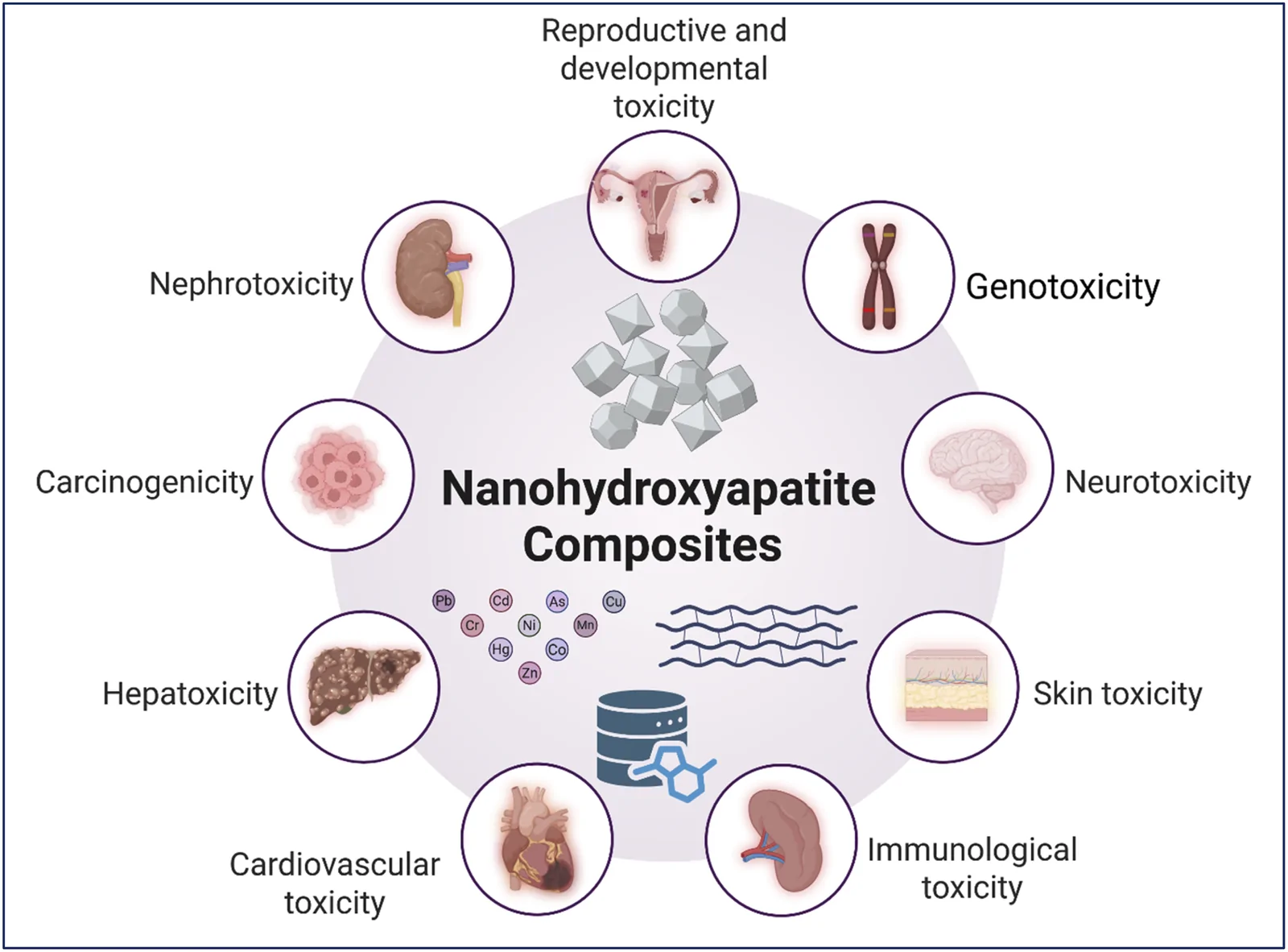
Schematic illustration of nHAp composites, their sources, composite components, main biomedical applications, and important toxicity issues. The figure is a summary of the multifunctional integration of nHAp with polymers, metals and ceramic materials, as well as the major toxicity-associated responses such as reproductive toxicity, cytotoxicity, genotoxicity, immunotoxicity, cardiovascular toxicity, *etc.* (the figure was created using BioRender.com).

The overall concept and toxicity concerns of nHAp composites for biomedical applications are shown in Fig. 1. The nanoscale size, high surface area, increased bioactivity, improved cellular interaction, and resorbability of nHAp materials make them ideal for incorporation with metallic particles, ceramics and polymeric materials to produce multifunctional composite systems for bone tissue engineering, drug delivery, dental applications, implant coatings, and antimicrobial platforms. Although the biomedical potential of nHAp composites is promising, there is growing evidence that nHAp composites can trigger a number of toxicity-related responses depending on the particle characteristics, composite composition, dosage, degradation behaviour and biological exposure conditions. These toxicity issues encompass cytotoxicity due to decreased cell viability, oxidative stress due to the production of ROS, genotoxicity due to DNA damage, immunotoxicity due to inflammatory response, dose- and shape-dependent biological effects, ion release-related toxicity, and potential *in vivo* biodistribution and organ-related complications.

Table 1 shows the comparative overview of conventional HAp and nHAp in terms of their physicochemical and biological properties. The particle size of nHAp is much smaller than that of bulk HAp, and the specific surface area is much larger, which leads to higher surface reactivity, protein adsorption, cell interaction and bioactivity. The nanoscale features enhance the adhesion of osteoblasts and the potential for bone regeneration, making nHAp highly suitable for advanced biomedical applications. But the increased surface activity and potential for internalization by cells may also increase nanosafety concerns in certain exposure scenarios. The comparison emphasizes the pros and cons of the nanoscale transformation of hydroxyapatite.

**Table 1:** Physicochemical and biological characteristics of HAp and nHAp for biomedical applications and toxicity behaviour

Parameter	Hydroxyapatite (HAp)	Nanohydroxyapatite (nHAp)	Consequence	References
Particle size	∼1–10 µm	∼20–100 nm	Reduced particle size increases surface reactivity and biological interaction	30 and 31
Specific surface area	∼2–5 m^2^ g^−1^	Frequently exceeds 100 m^2^ g^−1^	Higher surface area promotes protein adsorption and osteoblast attachment	32
Crystallinity	Higher crystallinity	Lower to moderate crystallinity	Lower crystallinity enhances biodegradability and bioactivity	31
Protein adsorption	Relatively lower	∼40–50% higher osteoblast adhesion/proliferation and significantly increased vitronectin adsorption compared to conventional HAp	Enhanced cell adhesion and proliferation	30
Mechanical brittleness	Brittle with fracture toughness ∼0.7–1.2 MPa m^1/2^	Improved toughness up to ∼1.5–2.5 MPa m^1/2^	nHAp is generally used in reinforced composite systems for better mechanical stability and load transfer	33 and 34
Toxicity concern	Cell viability >90%	Cell viability ∼60–80% at higher concentrations (>100–200 µg mL^−1^), depending on particle size, morphology, and exposure duration	Strongly influenced by dose, morphology, surface characteristics, and composite composition	27 and 35

Thus, beyond previous reviews, which majorly focused on the synthesis, physicochemical properties and biomedical applications of HAp or nHAp composites, the present review sheds light on the critical and unexplored area of nHAp composites, such as toxicity and bio-safety. This review systematically correlates physicochemical properties of nHAp composites with biocompatibility and application-specific safety regulations. The special emphasis was given on how particle size, surface morphology, crystallinity, *etc.*, affect the potential toxicity and biological performance of nHAp. Furthermore, the review highlights current limitations, inconsistencies among published studies, and the urgent need for standardized toxicity assessment protocols to facilitate regulatory acceptance and clinical translation of nHAp-based biomaterials. The review follows an organized structure that begins with the fundamentals of HAp and nHAp composites in Section II, followed by an outlook of different additives (polymeric, metallic, carbon and ceramic-based). Subsequently, the major toxicity mechanisms, including cytotoxicity, oxidative stress, genotoxicity, immunotoxicity, and *in vivo* toxicity, are critically evaluated together with the key physicochemical factors governing these biological responses (Section III). The review further correlates these toxicological findings with application-specific biosafety requirements in bone tissue engineering, implant coatings, antimicrobial systems, drug delivery, dental applications, imaging, and cancer therapy, highlighting optimized formulations and concentration-dependent toxicity (Section IV). Finally, the review identifies the existing knowledge gaps, translational challenges, and future research priorities required for the safe clinical development and regulatory acceptance of multifunctional nHAp-based biomaterials (Sections V and VI). Overall, the structure and focus of the review are essential, which provides researchers with a quick and critical overview of the nHAp formulations and characteristics for controlled toxicity procedures and long-term safety regulations.

## 2. Nanohydroxyapatite composites

### 2.1 Nanohydroxyapatite

The calcium phosphate-based mineral nHAp is often used in regenerative medicine due to its large surface area and bioactivity.^36^ In addition to being a calcium ion source and biophysical signaler for the development of growth cones, axon guidance, and neuronal synapse processes, nHAp is a mineral phase that can produce nanotopographies for extracellular matrix contraction and surface wettability appropriate for biomedical applications.^37^ A self-assembling hydroxyapatite structure with at least one dimension that is no larger than 100 nm in three dimensions is called nHAp. The distinctive characteristics of this nanomaterial are its ultrafine structure and strong surface activity. nHAp can appear in various shapes like rod, spherical, sheet and needle. Among all these shapes, needle and rod-shaped nHAp are most prevalent.^20^ To create nHAp, various synthesis techniques have been developed, each designed to produce specific particle sizes and shapes, including electrodeposition, electrophoretic deposition, sol–gel, thermal spray, micro arc, physical vapour deposition, *etc.*^38^ Each technique offers unique benefits and drawbacks that significantly impact nHAp's physical characteristics and applicability for certain uses, including as drug delivery systems and tissue engineering (Table 2).

**Table 2:** A comparative overview of various techniques utilized for surface modifications of nHAp. Reproduced with permission from ref. 38. Copyright 2024 IOP

Technique	Principle	Advantages	Limitations	Typical applications
Electrodeposition	Involves the movement of ions in an aqueous electrolyte under an applied electric field using conductive electrodes	Can be performed at ambient conditions, enables thick and uniform coatings over extended durations	Direct current deposition may produce non-uniform coating thickness and anode passivation at low current density	Fabrication of biosensors, microreactors, and photonic devices
Electrophoretic deposition	Relies on the migration of charged particles suspended in an organic medium toward conductive substrates	Suitable for porous and geometrically complex substrates	High ionic conductivity can decrease particle mobility and trigger unwanted side reactions	Energy storage systems, field emission devices, MEMS, and solar cells
Sol–gel method	Based on hydrolysis and condensation of reactive soluble precursors to form gel networks	Provides excellent surface coverage and allows coating of complex-shaped components	Limited compressive strain resistance and possible toxicity associated with chemical precursors	Sensors, protective coatings, flame-retardant and UV-protective textile finishing
Thermal spray	Utilizes molten or semi-molten particles propelled toward the substrate surface	Produces high-quality coatings and accommodates a wide particle-size range	Less effective for substrates with blocked or intricate geometries	Thermal barrier coatings and biomedical implant coatings
Micro-arc oxidation	Employs plasma-assisted sparking discharge generated in electrolytic media under DC power supply	Improves hardness and corrosion resistance of coated surfaces	Process complexity and formation of defects such as micropores and cracks	Tribological, biomedical, dielectric, and photovoltaic coatings
Physical vapour deposition (PVD)	Involves vaporization of materials using energetic ion/electron beams followed by thin-film condensation	Environmentally benign process with highly durable and abrasion-resistant coatings	Requires high vacuum and elevated operating temperatures	Decorative coatings, cutting tools, and anisotropic glass coa

In recent years, nHAp has attracted tremendous attention due to its significant physicochemical and biological properties compared to the bulk or microscale HAp. The nanoscale dimensions of nHAp closely resemble the mineral phase of natural bone, leading to increased bioactivity, increased surface reactivity, increased protein adsorption and increased osteoblast adhesion and proliferation. The properties of nHAp support rapid bone regeneration, good tissue integration, and good osteogenic activity, which makes it very promising for bone tissue engineering, coating of implants, drug delivery and applications in regenerative medicine. Furthermore, the nanoscale surface roughness and high surface area of nHAp promote better cell–material interactions and biological responses at the tissue interface. Despite these benefits, however, nHAp is generally not mechanically strong and brittle, which limits its use in load-bearing biomedical applications alone. For this reason, different reinforcing agents have been added to nHAp to create multifunctional composite systems, which exhibit enhanced mechanical stability, biological performance, and application-specific properties, discussed in the subsequent section.

### 2.2. Various fillers for n-HAp composites

Composites are new materials created by physical or chemical processes from the combination of two or more materials having distinct features. Clinical uses of simple n-HAp biomaterials are limited by issues such as poor mechanical strength, fatigue failure, and inadequate fracture toughness.^39^ To enhance its performance, n-HAp has been mixed with other biological materials in a number of investigations.

#### 2.2.1 Polymeric nHAp composites

Chitosan (CS), silk fibroin (SF), polylactic acid (PLA), polycaprolactone (PCL), *etc.*, are considered interesting polymers for various biomedical utilizations due to their improved bioactivity and non-toxicity.^40–42^ CS and SF are natural polymers, derived from chitin and silk, respectively.^43,44^ Despite CF and SF's widespread usage in bone tissue engineering in contemporary medicine, their poor mechanical nature and instability have restricted their use in load-bearing areas. Similarly, PLA and PCL's applicability have been restricted due to their hydrophobic surface, lack of osteoinductivity, sluggish rate of degradation, and acidic hydrolysis products that might cause inflammation at the scaffold implantation site.^45,46^ As a result, the combination of these polymeric reinforcements with nHAp can enhance the osteoconductivity and surface properties.

Eydi and co-workers^47^ synthesized nHAp with CS and varying amounts of lithium for human dental pulp stem cells (hDPSCs). The scaffolds facilitated the attachment of hDPSCs, as shown by biological experiments; at Day 14, the Li/n-HA/Ch-100 group maintained considerably high proliferation of cells with *p* < 0.01. Remarkably, osteogenic differentiation was significantly improved by lithium-loaded scaffolds; in particular, the Li/n-HA/Ch-200 group had higher alkaline phosphate activity (*p* < 0.0001). This study concluded Li/n-HA/Ch scaffolds as viable options for bone tissue engineering applications.

**Fig. 2:**
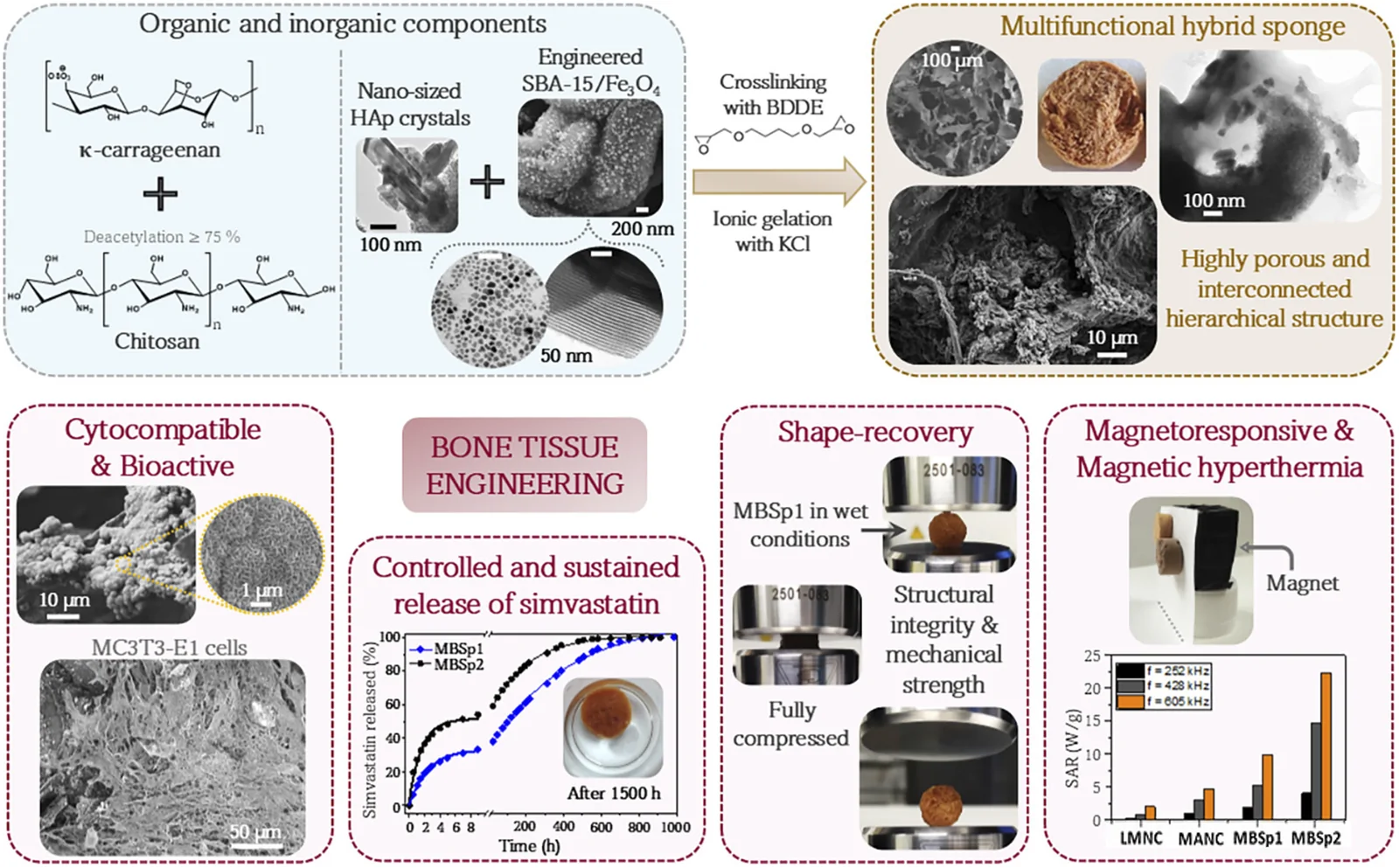
Schematic illustration and multifunctional properties of BDDE-crosslinked chitosan/κ-carrageenan hybrid sponges containing nHAp crystals and engineered SBA-15/Fe_3_O_4_ composites for bone tissue engineering applications. The fabricated porous and hierarchically interconnected sponges showed good cytocompatibility and bioactivity, improved cell adhesion and proliferation, controlled and sustained simvastatin release, shape-recovery behaviour under wet compression and magnetoresponsive hyperthermia performance under an external magnetic field. The integration of nHAp enhanced the osteogenic potential and biological interactions, whereas SBA-15/Fe_3_O_4_ composites offered hierarchical porosity, drug-loading capacity, and magnetic functionality, making them ideal for creating multifunctional platforms for advanced bone regeneration applications. Reproduced with permission from ref. 48. Copyright 2026 Elsevier.

Vargas-Osorio *et al.*^48^ developed a sponge using nHAp, CS, crosslinked with 1,4-butanediol diglycidyl ether (BDDE) and SBA-15/Fe_3_O_4_. The encapsulation of SBA-15/Fe_3_O_4_ particles attributed to high loading (≥150 mg g^−1^) of simvastatin (a medication that may improve bone regeneration) and its controlled release over prolonged durations (≥30 days) due to its significant surface roughness and porosity. Furthermore, under a magnetic field of 28 mT at various kHz, these designed composites achieved specific absorption rates (from 1.82 W g^−1^ to 22.44 W g^−1^), giving them the capacity to regulate the heat response. Assessments of cytocompatibility employing human osteosarcoma (MG-63), preosteoblast (MC3T3-E1), and mouse macrophage (RAW 264.7) cell lines showed ≥80% viability in all scenarios, with the maximum proliferation in direct contact. These adaptable and cooperative sponges have potential uses in bone tissue engineering (Fig. 2).

#### 2.2.2 Metallic nHAp composites

Antibacterial capacity of nHAp is another important characteristic for bone grafts and dental implant applications to avoid infections after surgery.^49^ The most effective method for enhancing HAp's antibacterial activity is doping with metal ions.^50,51^ Several metal-doping ions, including magnesium, silver, zinc and copper ions, were used in earlier studies to increase the antibacterial activity of HAp. Because of its strong antibacterial properties, silver has been investigated the most.^52,53^

**Fig. 3:**
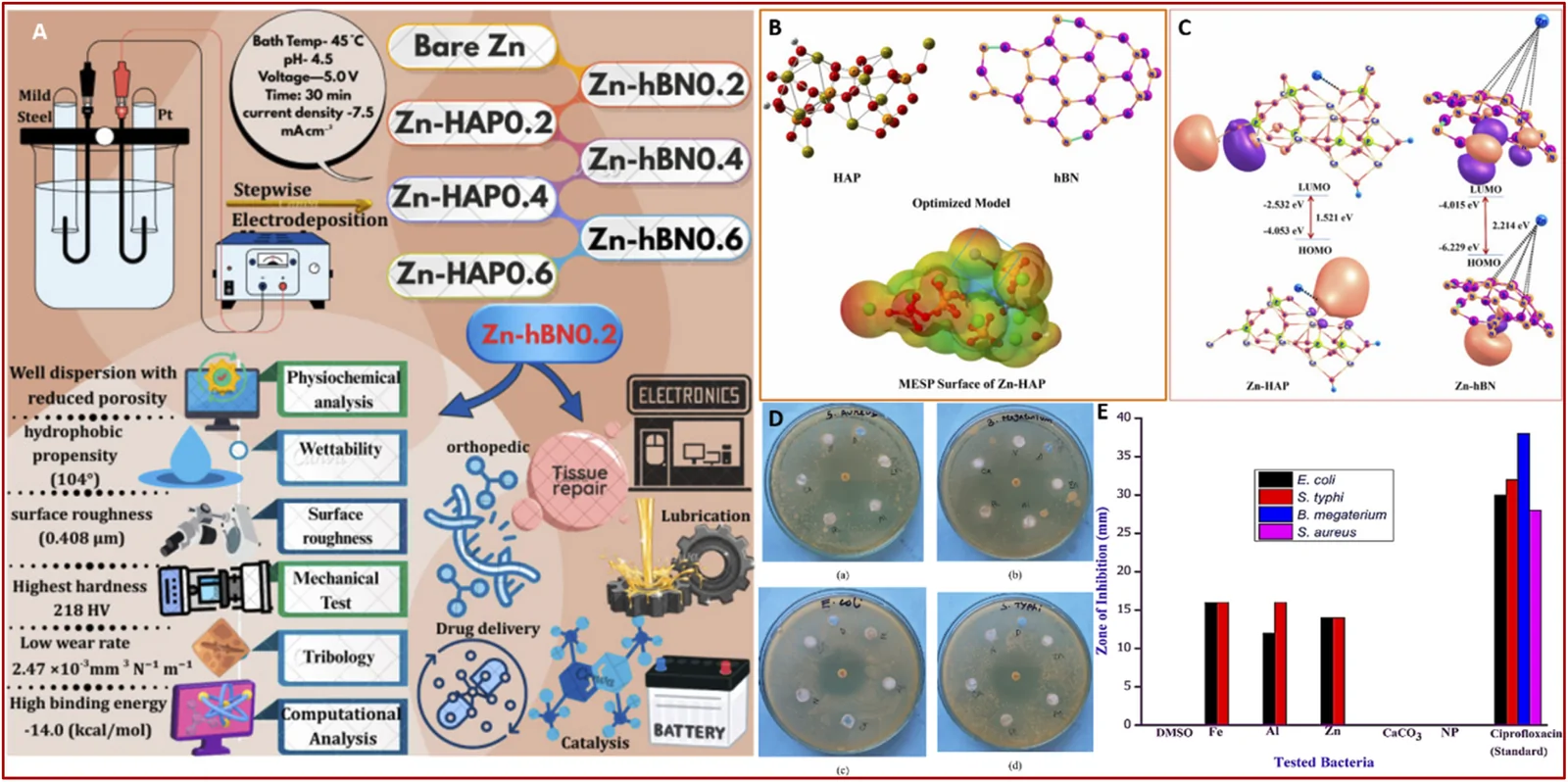
(A) Diagrammatic depiction of the sample preparation procedure and characterization techniques of Zn, HAp and hBN-based samples (B) at the B3LYP/6-31G (d, p) level of theory, the optimized structures of the HAp (top left), hBN (top right), and MESP plot of the Zn-HAp Composite (bottom) models. The Ca, O, P, and H atoms are represented on the MESP surface by the colors green, red, orange, and white, respectively. (C) The 3D-Isosurface of HOMO and LUMO Zn-HAp (left) and Zn-hBN (right) maps complexes at the theoretical level of B3LYP/6-31G (d, p). Reproduced with permission from ref. 54. Copyright 2025 Elsevier. (D) Antimicrobial zones of inhibition of various metal-HAp samples against Gram-negative (c) *E. coli*, (d) *S. Typhi* and Gram-positive (a) *S. aureus*, (b) *B. megaterium* bacteria samples. (E) Fe, Al, and Zn ions' antimicrobial activity when hydroxyapatite and ciprofloxacin are used as positive controls. Reproduced with permission from ref. 55. Copyright 2024 Cell Press (Open Access CC by 4.0).

Phogat *et al.*^54^ synthesized nHAp with Zn metal as well as hexagonal boron nitride (hBN) separately, using the electrodeposition technique. With the highest microhardness, adhesion strength, and improved wear resistance (218 HV, 38.1 MPa and wear rate 2.47 × 10^−3^ mm^3^ N^−1^ m^−1^, respectively), the Zn-hBN0.2 composite coating emerged as a potential coating for industrial applications. The synergistic impact of the enhanced interfacial interaction between Zn and hBN and the intrinsic solid lubrication of hBN greatly improved the tribological performance, according to the combined experimental and computational studies (Fig. 3A). Density functional theory (DFT) simulations were used to quantify interfacial energetics and electrical characteristics in order to clarify the underlying processes. Consistent with experimental hardness and roughness results, the Zn-hBN system showed more stability than Zn-HAp (binding energy: −14.0 kcal mol^−1^, Egap: 2.214 eV for Zn-hBN, while for Zn-HAp, binding energy: −9.3 kcal mol^−1^, Egap: 1.521 eV) (Fig. 3B and C). Overall, this study concluded Zn, HAp and hBN-based coatings for various industrial applications.

Because HAp has a significant ion exchange capacity and a crystal structure made up of Ca^2+^, OH^−^, and (PO^4^)^3-^, the Ca^2+^ ions in its crystal lattice may be replaced by other ions, such as Al^3+^, Fe^3+^, Zn^2+^, Ni^2+^, Na^+^, *etc.*, to alter different aspects of synthetic HAp.^56,57^ Additionally, magnetic metal ions like Fe^3+^ and Co^2+^ were incorporated into HAp nanoparticles and used for targeted drug delivery, MRI, cell separation, and hyperthermia. Al-enriched HAp produced highly anisotropic particles and reduced crystallinity, which increased MG-63 cell proliferation.^58^ The strong antibacterial activities of Ni^2+^-doped silicate hydroxyapatite against *P. aeruginosa* and *E. coli* were confirmed by another investigation.^59,60^ In recent research, Habib and team^55^ have presented a conventional wet chemical precipitation approach for the manufacture of several metal-doped nHAp. In comparison to pure HAp, metal-doped hydroxyapatite HAp displayed significant antibacterial properties against *S. typhi* and *E. coli*. The metal-doped HAp exhibited an inhibitory zone that varied between 14 and 16 mm. Among all the ions studied, the Fe ion showed the largest inhibitory zone, measuring 16 mm (Fig. 3D and E). The results showed that all produced HAp samples had the potential to be dependable biomaterials.

Various metal ions like Ag, Zn, Sr, Si, Ce, Mg, *etc.*, play an important role in governing biocompatibility and toxicity of nHAp composites for several biomedical applications.^61–63^ Addition of Ag and Zn into HAp offers noteworthy antimicrobial activity and osteoblast proliferation through controlled release of Ag^+^ and Zn^2+^ ions, but excessive release of these ions can also damage cell proliferations to the generation of ROS (Table 3). Strontium is the most promising dopant for bone regeneration as it promotes osteoblast differentiation and enhances osteogenesis. Mg and Si enhance mineralization and regulate enzymatic activities through the sustained release of respective ions. Cerium shows a reversible Ce^3+/^Ce^4+^ redox reaction and exhibits anti-inflammatory properties, while an excess amount of Ce resulted in adverse effects on cellular response. Thus, a systematic mechanism and optimized study are required for different ion-doped nHAp composites for balanced biocompatibility and bio-safety.

**Table 3:** A comparative overview of the biological benefits, limitations and related mechanisms for metal ion-doped nHAp composites

Dopant ion	Major biological benefits	Potential toxicity or limitations	Underlying mechanism	References
Ag^+^	Strong antibacterial activity	Excess Ag ion release can damage cellular response and increase ROS	Ag ions release disrupts bacterial membranes, but excessive release induces oxidative stress	61 and 64
Zn^2+^	Promotes osteoblast proliferation, mineralization and antibacterial activity	Excess Zn^2+^ may impair cell viability through ion imbalance	Regulation of osteogenic enzymes and Zn-dependent signalling	62 and 65
Sr^2+^	Enhances osteogenesis, angiogenesis	Very high Sr content may alter HAp crystallinity	Regulating enzymatic activity	56 and 66
Mg^2+^	Improves cell adhesion, proliferation and mineralization	Excess Mg may accelerate degradation and alter mechanical stability	Enzyme cofactor	67 and 68
SiO_4_^4−^	Stimulates collagen synthesis, apatite formation and osteogenic differentiation	Excess silicate release may alter local pH and degradation behaviour	Sustained silicate release promotes biomineralization	60
Ce^3+^/Ce^4+^	Antioxidant activity, ROS scavenging and anti-inflammatory behaviour	High Ce loading may affect crystallinity and dose-dependent cytocompatibility	Reversible Ce^3+^/Ce^4+^ redox cycling	63 and 69
Cu^2+^	Antibacterial activity and angiogenesis	Excess Cu^2+^ promotes oxidative stress and cytotoxicity	Redox-mediated	70 and 71

#### 2.2.3 Ceramic and carbon-based nHAp composites

**Fig. 4:**
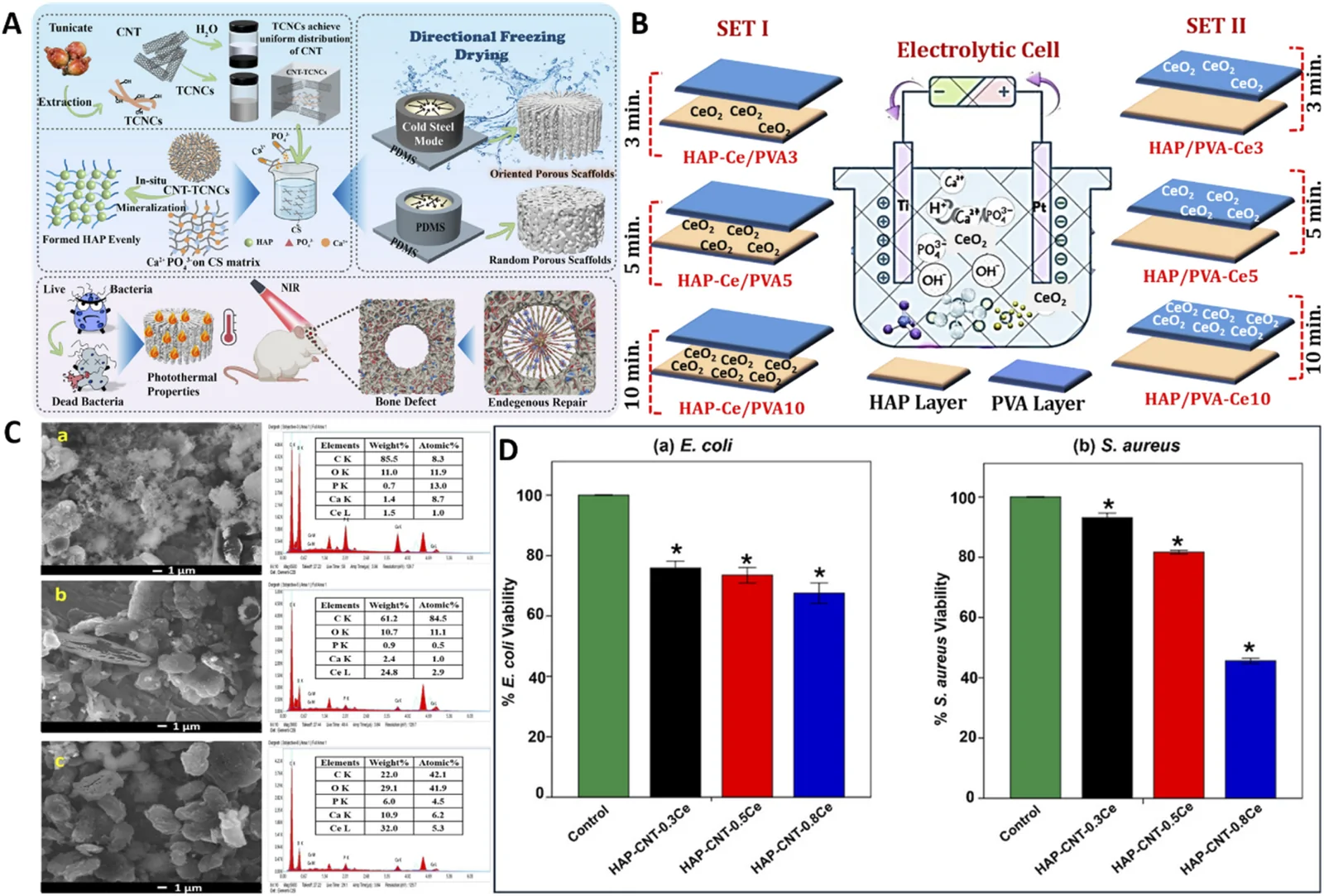
(A) Diagrammatic representation of the radial composite scaffold's construction and operation. Reproduced with permission from ref. 72. Copyright 2026 Wiley. (B) Diagrammatic illustration of the electrochemical deposition process used in the experimental setup to create a HAp double-layer composite coating on a Ti alloy substrate. Reproduced with permission from ref. 73. Copyright 2025 Elsevier. (C) (a) HAp-CNT-0.3Ce, (b) HAp-CNT-0.5Ce, and (c) HAp-CNT-0.8Ce, respectively, show the surface morphology of the HAp-CNT-Ce composite coatings. (D) Shows the quantitative assessment of the antibacterial effectiveness of electrochemically deposited HAp-CNT-0.3Ce, HAp-CNT-0.5Ce, and HAp-CNT-0.8Ce composite coatings against (a) *E. coli* and (b) *S. aureus*, where * denotes the significant difference between the bacterial viability on all of the HAp-CNT-Ce coatings and the control (titanium substrate). Reproduced with permission from ref. 63. Copyright 2025 Wiley.

Ceramic and carbonaceous particles reinforced composites have gained a lot of research interest owing to their remarkable mechanical strength, thermal stability, and resistance to corrosion and wear. A nano-homogeneous, radially orientated hybrid scaffold for endogenous bone regeneration was created by Yu *et al.*^72^ using directional freeze-casting technology, drawing inspiration from the anisotropic design of real bone. In order to obtain uniform dispersion of carbon nanotubes (CNT) inside a chitosan (CS) matrix, tunicate nanocellulose (TCNC) and HAp were used as an effective dispersant, mechanical and biological reinforcer. With an antibacterial rate of more than 98% against *S. aureus*, the scaffolds' potent photothermal antibacterial capacity was enhanced by the addition of CNT. After three months of implantation, the oriented scaffold demonstrated improved osteogenic efficacy in a rat cranial defect model, with bone mineral density reaching 0.688 g cm^−3^. Overall, the study highlighted the significance of nano-homogeneous, anisotropic designs in directing tissue restoration (Fig. 4A).

Phogat *et al.*,^63,73^ in recent research, encapsulated cerium oxide (CeO_2_) and carbon nanotubes (CNT) with nHAp using the electrodeposition method for bone tissue engineering. In the first work, they investigated the impact of ceria (CeO_2_) additive on the double-layered coating composed of hydroxyapatite/polyvinyl alcohol (PVA) (Fig. 4B). When compared to longer times or inverted CeO_2_ combinations, the optimized 3 minute deposition produces better results due to strong interfacial strengthening. HAp-Ce/PVA3 exhibits better Vickers hardness and adhesion strength (1600 HV and 58 MPa, respectively), as well as improved crystallinity and refined grain size. Improved bioactivity was shown by biocompatibility experiments, which verified >75% cell viability. On the other hand, in another research, they have added CeO_2_ in varying concentrations (0.3–0.8 wt%) to the HAp-CNT coatings. The morphological analysis of the HAp-CNT-Ce coatings (Fig. 4C a–c) shows that Ce and CNT have been uniformly assimilated into the HAp matrix, resulting in a composite interface on the substrate that is both mechanically cohesive and architecturally integrated. HAp-CNT-0.8Ce coating showed improved hardness, adhesion strength and antimicrobial activity against *E. coli* and *S. aureus* (354 HV, 52 MPa, reduced viability of 67.5% and 45.6%, respectively) (Fig. 4D). This performance was due to the refined crystallite size (20 nm) for HAp-CNT-0.8Ce. In the context of translational biomedical engineering, this functional coating was referred to as an advanced material for bone repair applications.

In general, the use of various reinforcing materials like polymers, metals, ceramics and carbonaceous materials has greatly broadened the functional capabilities of nHAp-based composites for biomedical applications. These reinforcing components not only enhance the mechanical stability and physicochemical properties of nHAp systems, but also impart other functionalities such as antimicrobial properties, electrical conductivity, magnetic responsiveness, and controlled drug delivery behaviour. The presence of such materials can also modify the biological interactions, degradation, cellular uptake and long-term biosafety of the composites, however. The biological response of particles depends on many factors, including particle size, morphology, surface chemistry, concentration, and ion release behaviour, in many cases. Thus, to ensure safe biomedical translation, a thorough understanding of the toxicity behaviour of nHAp composites is crucial, despite their promising therapeutic and regenerative properties. The next section covers the key factors that affect the toxicological behaviour of nHAp composites and the major toxicity concerns.

Table 4 offers a comparative outlook on the polymeric, metallic, carbon and ceramic-based additives in nHAp matrix. The table summarizes the key findings of quantitative performance, clinical activity and associated toxicity concerns of nHAp composites. It highlights how parameters like cell viability, antibacterial activity, mechanical properties, drug loading and toxicity are interlinked to each other. The table shows that the type of reinforcing agent can alter quantitative and qualitative behaviour and thus, the toxicity profile of nHAp composites, which provides a fundamental framework for the selection of appropriate additives with a balance of theranostic ability and long-term biosafety of nHAp composites.

**Table 4:** Comparative analysis of different reinforcing agents used in nHAp composites for biomedical applications and their influence on biological performance and biosafety

Reinforcing agent	Composites	Optimized composition/key outcomes	Major biomedical advantages	Potential toxicity	Ref.
Polymeric	Li/nHAp/chitosan scaffold	Li/nHA/Ch-100 showed significantly higher hDPSC proliferation (*p* < 0.01); Li/nHA/Ch-200 exhibited highest ALP activity (*p* < 0.0001)	Enhanced osteogenic differentiation, excellent cell attachment and bone regeneration	Excess Li loading or polymer degradation products may alter local cellular response; optimization of polymer composition is essential	47
	BDDE-crosslinked chitosan/κ-carrageenan/nHAp/SBA-15/Fe_3_O_4_ sponge	Simvastatin loading ≥150 mg g^−1^; sustained release ≥30 days; SAR = 1.82–22.44 W g^−1^ (28 mT); cell viability ≥80% (RAW264.7, MG-63, MC3T3-E1)	Controlled drug delivery, magnetic hyperthermia, high cytocompatibility	Long-term biosafety depends on magnetic particle concentration, degradation behaviour and drug release kinetics	48
Metallic	Zn-hBN/nHAp coating	Hardness 218 HV; adhesion strength 38.1 MPa; wear rate 2.47 × 10^−3^ mm^3^ N^−1^ m^−1^; DFT binding energy −14.0 kcal mol^−1^	Improved wear resistance, mechanical stability and coating durability	Metal-ion release should be controlled to avoid excessive ROS generation and dose-dependent cytotoxicity	54
	Fe, Zn, Cu, Ag-doped nHAp	Antibacterial inhibition zone 14–16 mm; Fe-doped nHAp showed the largest inhibition zone (16 mm)	Strong antibacterial activity for implant coatings and bone grafts	Excess dopant concentration may increase ion release and oxidative stress despite enhanced antibacterial efficacy	55
Ceramic/carbon-based	CNT/TCNC/CS/nHAp scaffold	Antibacterial efficiency >98% against *S. aureus*; bone mineral density 0.688 g cm^−3^ after 3 months	Excellent osteogenesis, anisotropic bone regeneration and photothermal antibacterial activity	CNT dispersion and long-term persistence should be carefully controlled to minimize inflammatory reactions	72
	CeO_2_/PVA/nHAp coating	Hardness 1600 HV; adhesion strength 58 MPa; cell viability >75%	Excellent mechanical reinforcement with good bioactivity	Excess ceramic loading may influence degradation behaviour and coating brittleness	73
	CeO_2_-CNT/nHAp coating	Hardness 354 HV; adhesion strength 52 MPa; crystallite size 20 nm; bacterial viability reduced by 67.5% (*E. coli*) and 45.6% (*S. aureus*)	High antimicrobial activity with improved mechanical properties	Cerium concentration and CNT content should be optimized to balance antibacterial efficacy and cytocompatibility	63

## 3. Toxicity of n-HAp composites and factors influencing toxicity

The nanohydroxyapatite composites have been a subject of great interest in biomedical engineering because of their osteoconductivity, biomimetic properties and multifunctional behaviour. Although the nanoscale size and high surface reactivity of nHAp have been shown to have therapeutic potential, there is growing evidence that nHAp can also elicit unwanted biological responses under specific exposure conditions.^22,74^ The toxicity of nHAp composites is very complex and is influenced by several physicochemical and biological parameters such as particle size, morphology, concentration, surface charge, crystallinity, tendency to aggregate, dopants, degradation behaviour and exposure duration.^74^ Furthermore, the addition of reinforcing materials like metals, ceramics, polymers and carbonaceous materials can further alter the biological interactions and toxicological behaviour of these composite systems.^75,76^ Many studies have shown that the toxicity of nHAp composites is dose-dependent and is highly dependent on the morphology and surface properties of the particles. Many reports indicate that lower concentrations are biocompatible, but higher concentrations or longer exposure can cause oxidative stress, mitochondrial dysfunction, inflammation, DNA damage, and changes in cellular signalling pathways.^27,77^

### 3.1 Cytotoxicity

Cytotoxicity is among the most extensively investigated toxicological concerns associated with nHAp composites. In general, nHAp exhibits relatively good cytocompatibility at low to moderate concentrations; however, toxicity may arise at elevated concentrations or when particles possess highly reactive nanoscale morphologies. Cytotoxicity is commonly evaluated through cell viability assays, apoptosis analysis, studies of membrane integrity, and measurements of mitochondrial activity. Several studies reported that nHAp concentrations below approximately 50–100 µg mL^−1^ generally maintain cell viability above 80–90% in osteoblast and fibroblast cell lines.^78,79^ However, exposure at concentrations exceeding 100–200 µg mL^−1^ may significantly reduce cell proliferation and metabolic activity, depending on particle characteristics and exposure duration.^14,24,80^

Shi *et al.*^35^ demonstrated size-dependent toxicity of hydroxyapatite nanoparticles, where large sized nanoparticle (∼80 nm) induced apoptosis and reduced proliferation of osteoblast-like cells. One major mechanism responsible for cytotoxicity is mitochondrial dysfunction. Due to their nanoscale dimensions, nHAp particles can be internalized through endocytosis and accumulate within intracellular compartments. Excessive nanoparticle accumulation may disrupt mitochondrial membrane potential, impair ATP production, and activate apoptotic signalling pathways. Mitochondrial damage is often accompanied by increased production of ROS and activation of caspase-mediated apoptosis. In addition to particle size, cell behaviour was also influenced by the crystallinity and shape of nHAp particles. Prior research has shown that the behaviour of cultivated osteogenic cells is influenced by the crystallinity of calcium phosphate.^81,82^ Furthermore, shape ought to be a crucial factor in determining the biological impact of calcium phosphate particles. Two types of nHAp: rod-like and sphere-like, were found in the investigation by Shi *et al*.^35^ As reported by Zhao *et al.*,^83^ the cell experiment revealed that HAp with spherical nanocrystals exhibited more advantageous features for osteoblasts than rod-like HAp. The sphere-like nano-HAp's advantageous impact on osteoblasts may be explained by its well-organized surface, which seems to be advantageous for filopodia protrusion. Cultures on amorphous calcium phosphate substrates exhibit more osteogenic differentiation than those on crystalline HAp substrates. Lower crystallinity, surface chemistry, and topography have been shown to promote cell adhesion and differentiation.^84–86^

**Fig. 5:**
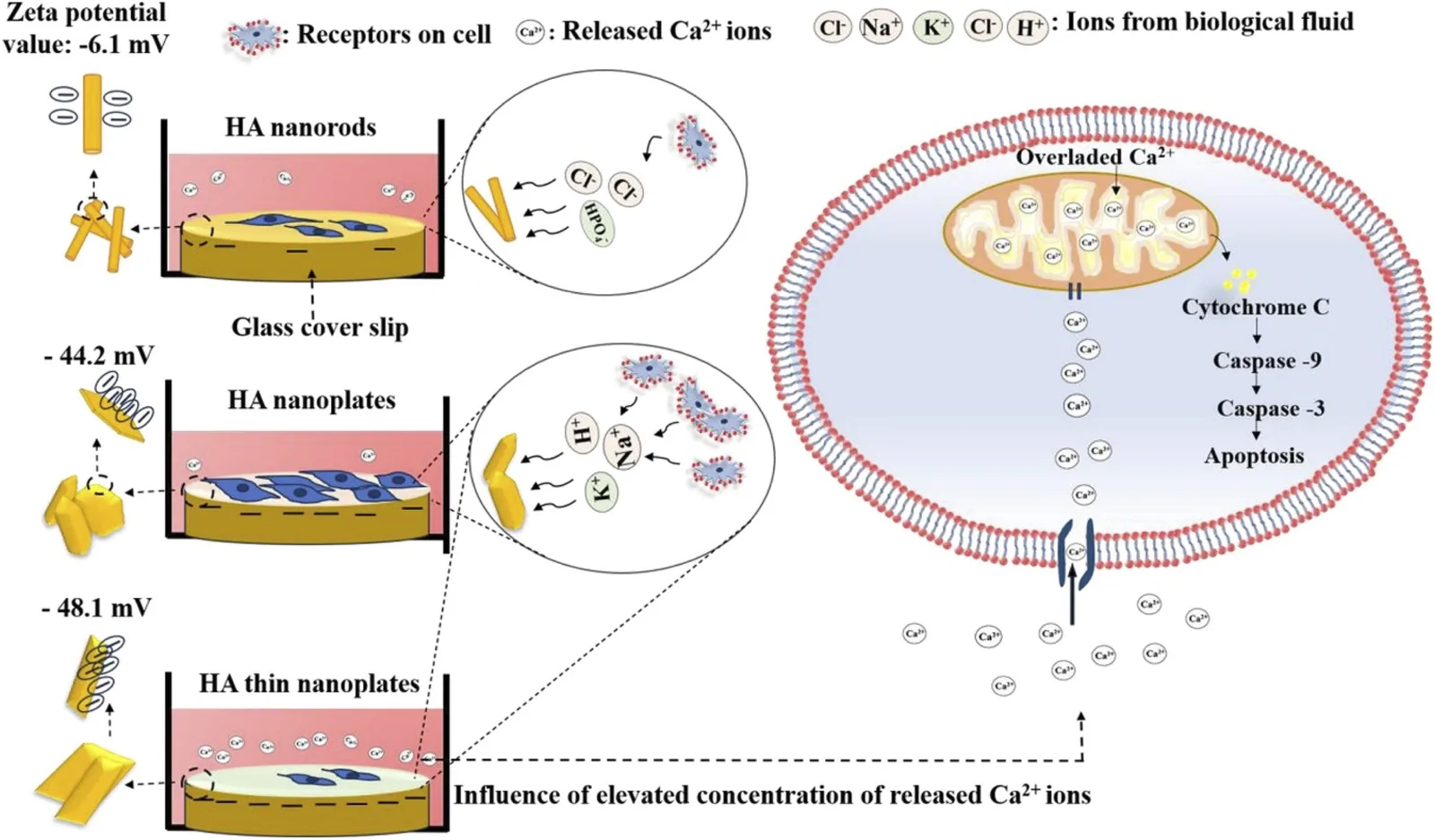
The schematic illustration showing how the release of Ca^2+^ ions and surface charge affect nHAp cellular response. Reproduced with permission from ref. 87. Copyright 2024 Elsevier.

The effect of the ion release behaviour of Ca^2+^ ions on biocompatibility and toxicity of nHAp was demonstrated by Kesarwani *et al.*.^87^ The nanoplates of HAp showed improved hemocompatibility (<5% hemolysis) with bioactivity and biosafety due to the controlled release of Ca^2+^ ions and moderate negative charge (−44.2 mV). Besides, the thin nanoplates exhibited the least cytocompatibility due to excessive calcium ions leaching, resulting in mitochondrial damage and cell death. On the other hand, nanorods having a lower surface charge (−6.1 mV) displayed moderate ion release and biological behaviour. Fig. 5 demonstrated that controlled ion release offered improved surface characteristics and biocompatibility, while excessive ion release resulted in less cytocompatibility and increased toxicity. Thus, overall optimized ion release kinetics is essential for a balance between biocompatibility and long-term bio-safety of nHAp composites.

**Fig. 6:**
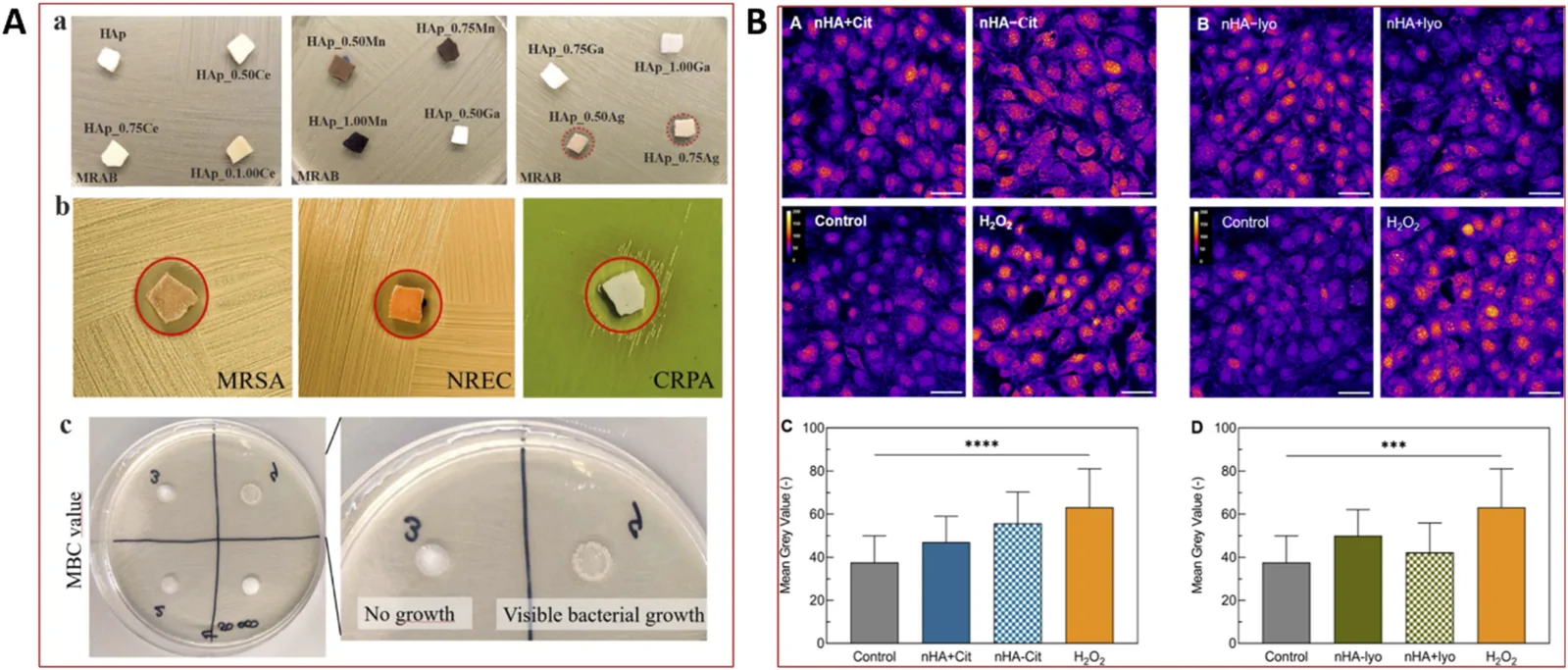
(i) (a) Representative agar plates of disc-diffusion test, without and with inhibition zones (circled in red); (b) representative agar plates of disc-diffusion test for methicillin-resistant *Staphylococcus aureus* (MRSA), non-resistant *E. coli* (NREC), and carbapenem-resistant *Pseudomonas aeruginosa* (CRPA) isolates with HAp_1.00Ag sample. Red highlights indicate the inhibition zone, where no bacterial growth is seen. (c) An illustration of how to calculate the minimal bactericidal concentration (MBC), which is the lowest concentration at which no discernible colony formation occurs. There are no bacterial colonies, even if the white smudge on the area marked as “no growth” is just the HAp powder^69^ (Open Access). (ii) ROS with nHAp exposure. MC-3T3 cells exposed to (A) nHAp synthesized at 40 °C with or without citrate, aged for five hours, and (B) nHAp synthesized at 60 °C with citrate, aged for five hours and agglomerated by lyophilization were shown in confocal live cell pictures. For ROS, cells were stained. Fire LUT is used to color images to show the intensity of ROS. (C and D) Staining intensity quantification. 50 µm is shown by the scale bar^88^ (Open Access).

In addition, metallic dopants such as Ag, Cu, and Zn incorporated into nHAp composites may further enhance cytotoxic effects due to ion release and oxidative stress generation. Ivankovic *et al.*^69^ studied the effect of various metals (Ce, Cu, Ga, Mn and Ag) with nHAp on cytotoxicity and antimicrobial activity. Except for Cu-substituted nHAp, which exhibited strong antibacterial activity against every tested bacterium, other materials were non-cytotoxic to cells. While Ga and Mn-substitution suggested possible antibacterial qualities against certain bacterial strains by preventing bacterial adhesion to its surface, Ce-substituted HAp had no antibacterial impact. Gram-negative bacteria adhered to the HAp surface less often than Gram-positive bacteria. It was estimated that as the amount of substituted Ag^+^ ions in nHAp grew, so did the antibacterial activity. Additionally, samples with the highest Ag^+^ content (1.00 mol%) demonstrated comparable antibacterial activity among tested bacteria, with the highest activity towards carbapenem-resistant *Pseudomonas aeruginosa* (CRPA) and the lowest for carbapenem-resistant *Klebsiella pneumoniae* (KPC), whereas samples with the lowest Ag^+^ concentration (0.5 mol%) demonstrated superior antibacterial activity towards methicillin-susceptible *Staphylococcus aureus* (MSSA), multidrug-resistant *Acinetobacter baumannii* (MRAB), and CRPA bacteria in comparison to other bacterial isolates (Fig. 6i).

A recent study on the preparation of nanohydroxyapatite composites containing silver showed that the way the silver is incorporated is crucial to the antibacterial activity and cytotoxicity behaviour.^61^ The addition of Ag nanoparticles significantly improved the antibacterial activity of hydroxyapatite, but too much Ag^+^ release and aggregation of the nanoparticles were correlated with a decrease in mammalian cell viability. The formulations investigated showed that Agx/HAp composites prepared by reverse precipitation (method 2) showed the best antibacterial activity and biocompatibility, especially for *x* = 0.2–0.3. The study also found that poorly dispersed AgNPs caused higher stress responses in the cells, while homogeneous dispersion of AgNPs decreased aggregation-induced cytotoxicity. The results indicate that the toxicity of Ag-doped nHAp systems is highly sensitive to the concentration of Ag, the distribution of the nanoparticles, the behaviour of ion release and the incorporation method, which makes it crucial to control the surface of the nanoparticles to obtain a balance between antimicrobial activity and biosafety.

Moreover, the effect of degradation kinetics on the biocompatibility and toxicity of nHAp was evaluated by Salimi *et al.*^89^ They designed nHAp composites (using varying concentrations) with polyethersulfone (PES) and wollastonite (WS) using the solvent casting method. The nHAp composites showed a similar degradation rate with controlled release of dexamethasone. Additionally, all compositions exhibited improved biocompatibility with no detectable toxicity. This observation was supported by the controlled degradation kinetics to ensure no excessive ion release or adverse cellular response in the biological microenvironment. It was concluded from the study that a controlled degradation is crucial for long-term bone implants due to enhanced mechanical strength, good bioactivity and minimal risk of toxicity from degradation.

Thus, from the above-discussed section, a critical analysis can be given on the cytotoxicity of the nHAp composites. Most of the studies reported that nHAp can show good cytocompatibility at lower concentrations (50–100 µg mL^−1^). This optimized concentration cannot be considered a universal threshold value, while the toxicity is majorly dependent on the shape, size, crystallinity and synthesis routes. This highlights the need for standardized dose concentration and optimized testing protocols before the translational utility of nHAp composites.

### 3.2 Oxidative stress and ROS generation

One of the main mechanisms of toxicity of nanoparticles is oxidative stress. The high surface area and high surface reactivity of nHAp composites can promote the production of intracellular ROS, which can cause oxidative damage to proteins, lipids, and nucleic acids. There are several reports that the generation of ROS is greatly enhanced as the concentration of nanoparticles and the duration of exposure increase. Andrée *et al.*^88^ recently studied the impact of hydroxyapatite nanoparticle crystallinity and colloidal stability on cytotoxicity and oxidative stress behaviour. The authors showed that low-crystalline and highly aggregated nHAp particles triggered significantly higher stress responses in the cells than well-dispersed particles. In particular, unstable colloidal systems resulted in greater ROS-related cytotoxicity as a result of increased aggregation and sedimentation of particles on the cell surface. The study also found that highly crystalline and colloidally stable nHAp displayed significantly better cytocompatibility, with cell viability remaining above ∼80–90% at moderate exposure conditions, while cell viability was significantly reduced at moderate exposure conditions in the case of unstable nanoparticle suspensions. The lighter tint in Fig. 6ii A/B indicates that cells exposed to nHAp had more ROS staining than the untreated control. When compared to colloidally stable nHAp (nHAp + Cit), agglomerated nHAp without citrate (nHAp–Cit) produced stronger staining. Colloidally stable nHAp demonstrated a 1.3-fold greater signal compared to the control when the average nuclear signal intensity of ROS staining was measured, whereas agglomerated nHAp demonstrated a 1.5-fold and 1.2-fold higher signal intensity compared to the control and colloidally stable nHAp, respectively (Fig. 6ii C). The lighter color in the images (Fig. 6ii B) and the 1.2-fold higher signal intensity of cells exposed to colloidally stable nHAp compared to cells exposed to agglomerated nHAp (Fig. 6ii D) demonstrate that, for nHAp with similar crystallinity (nHAp–lyo and nHAp + lyo), the ROS signal was higher for colloidally stable nHAp compared to agglomerated nHAp (nHAp + lyo). These results indicated that generation of oxidative stress is strongly related to physicochemical stability and dispersion behaviour of nanoparticles, not just to the composition of the particles.

In another recent study, Wang *et al.*^90^ showed that the polydopamine-coated hydroxyapatite nanoparticles could effectively promote the intracellular production of ROS and calcium ion accumulation in tumor cells, which ultimately led to mitochondrial damage and apoptosis in tumor cells through ROS-mediated signalling pathways. The study revealed that surface-modified nHAp induced high expression of ROS and activated ROS-mediated JNK signalling, leading to mitochondrial dysfunction and apoptotic cell death. Importantly, the improved oxidative stress response was correlated with the increase in intracellular Ca^2+^ accumulation and mitochondrial membrane disruption. The results presented here indicate that the oxidative behaviour of nHAp systems can be significantly altered by surface engineering and coating techniques, and that the biological safety profile can be affected. Furthermore, overproduction of ROS can result in inflammatory responses and oxidative damage in the case of chronic exposure.

The available evidence clearly indicates that ROS generation is governed not only by nanoparticle composition but also by colloidal stability, aggregation state, and surface engineering. However, most investigations quantify oxidative stress using short-term *in vitro* assays, whereas persistent oxidative injury under chronic exposure remains poorly understood. Future studies should correlate ROS production with intracellular degradation, inflammatory biomarkers, and long-term mitochondrial function to establish clinically meaningful toxicity thresholds.

### 3.3 Genotoxicity and immunotoxicity

Genotoxicity is defined as the potential of a material to cause DNA damage, chromosomal abnormalities or mutagenic effects.^27,91,92^ Recent studies indicate that nanostructured hydroxyapatite could have genotoxic effects under certain circumstances. The genotoxic properties of nHAp composites are mainly linked to ROS-induced oxidative damage, internalization of nanoparticles and chronic exposure of cells. Another important factor that affects the clinical safety of nHAp composites is their interaction with the immune system. Immune cells, such as macrophages, quickly detect nanoparticles after implantation or systemic exposure. The activation of macrophages is a key factor in the inflammatory response, biodegradation behaviour, and outcomes of tissue healing.^93–95^

A detailed review of 71 genotoxicity tests of hydroxyapatite-based biomaterials published in 2024 (ref. 27) found that 20 of those tests showed positive genotoxic effects, meaning that genotoxicity is very sensitive to the characteristics of the nanoparticles and experimental conditions. Importantly, nHAp particles with rod-like or needle-like shapes were more often linked to DNA strand breaks, the formation of micronuclei, and chromosomal abnormalities than spherical particles. The review also pointed out that high exposure levels (above ∼100–200 µg mL^−1^) and long exposure times led to a higher risk of oxidative DNA damage caused by the overproduction of ROS. However, at low concentrations, well-dispersed spherical nanoparticles showed little genotoxicity and good cell viability. The study revealed that the morphology, aggregation behaviour, crystallinity, and surface modification of nHAp systems are critical factors that affect the genotoxic profile. The results indicate that a careful design of the nanoparticles is crucial to reduce the DNA damage and enhance the long-term biosafety of nHAp-based biomedical materials.

**Fig. 7:**
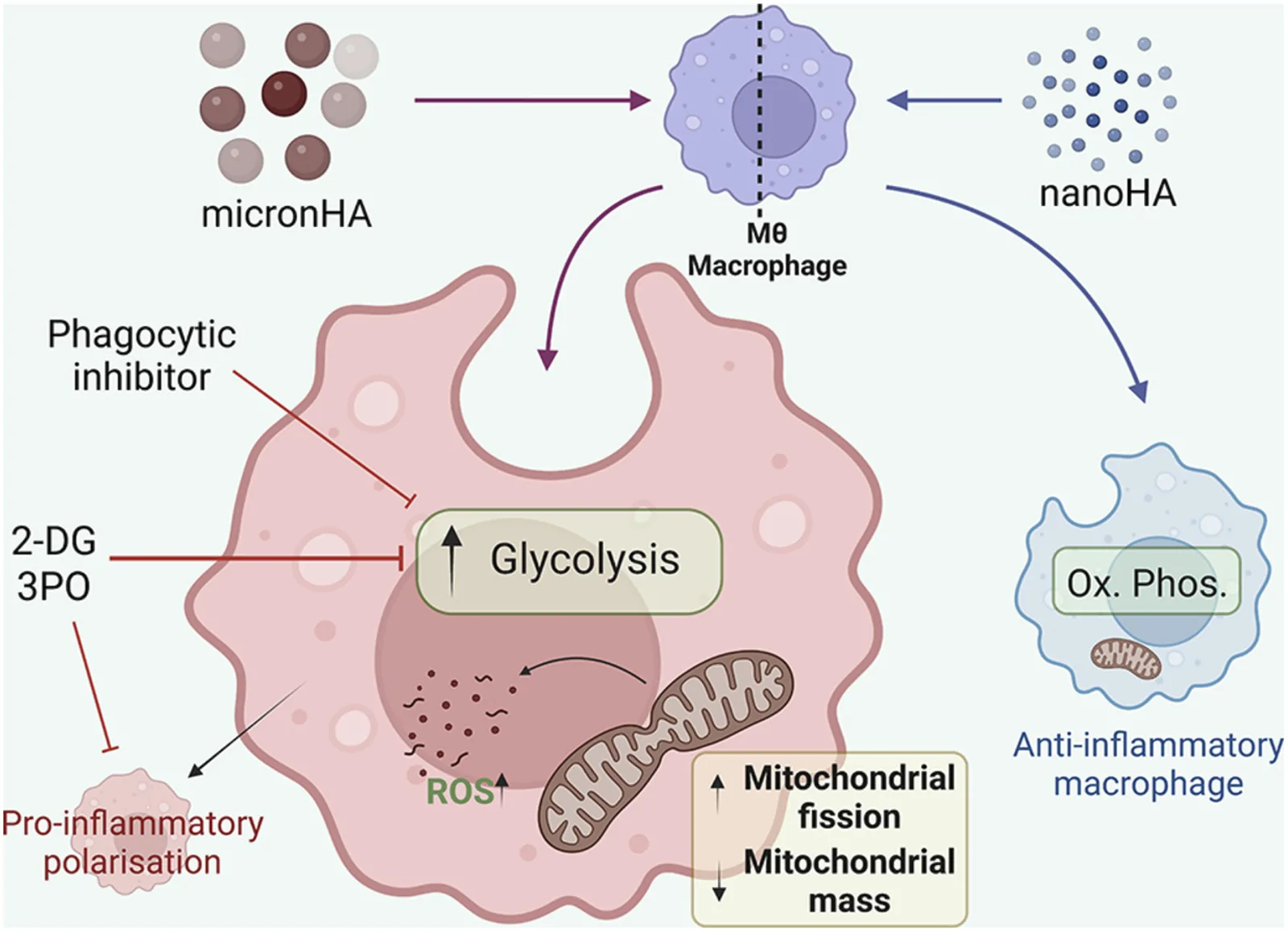
Schematic representation of the immunometabolic effect of hydroxyapatite particles on macrophages depending on their size^96^ (Open Access).

In the present study,^96^ it is shown that nHAp's immunological safety is closely linked to the particle size and the immune cell metabolic response. The authors demonstrated that the metabolic reprogramming of macrophages was much greater when exposed to micron-sized hydroxyapatite (micronHA) than to nano-sized hydroxyapatite (nanoHA). Macrophages use oxidative phosphorylation as the main source of energy production in resting conditions, whereas exposure to micronHA resulted in a shift towards glycolysis. Moreover, micronHA changed mitochondrial morphology and shifted towards a bioenergetic profile that was pro-inflammatory. Importantly, these changes were dependent on particle uptake by macrophages, and inhibition of glycolysis significantly reduced the pro-inflammatory response (Fig. 7). While this glycolytic activation is a normal short-term response to infection, it can lead to chronic inflammation, tissue damage, fibrosis, or negative post-implantation responses if stimulated continuously by biomaterial particles. Thus, the study indicates that hydroxyapatite-induced immunotoxicity is not solely correlated with direct cytotoxicity, but also with its capacity to induce inflammatory metabolic rewiring in immune cells, depending on the size.

The discussion on genotoxicity suggests that both genotoxicity and immunotoxicity are highly dependent on nanoparticle characteristics rather than being intrinsic properties of nHAp itself. Therefore, an integrated assessment combining DNA damage, immune activation, oxidative stress, and inflammatory biomarkers would provide a more reliable evaluation of nanosafety than isolated toxicity endpoints.

### 3.4 *In vivo* toxicity concerns

While many *in vitro* studies have shown good cytocompatibility of nHAp composites, *in vivo* toxicity is an important issue for clinical translation. Once the nanoparticles are taken up into the body or degraded, they can travel to various organs such as the liver, spleen, lungs, kidneys, and bone marrow.^97,98^ The impact of high-dose nano-hydroxyapatite exposure on the microcirculatory system *in vivo* was examined in recent research by Barros *et al*.^15^ According to the authors, topical nHAp treatment at a very high concentration of around 1 g kg^−1^ considerably reduced microcirculation and changed vascular behaviour. It's interesting to note that intravenous injection resulted in relatively smaller systemic biochemical changes, suggesting that exposure route and local nanoparticle concentration had a significant impact on toxicity. The research emphasized that because of nanoparticle accumulation and interaction with endothelial structures, excessive nHAp exposure may impair vascular homeostasis and microvascular function.

Another significant *in vivo* investigation showed that after systemic exposure, hydroxyapatite nanoparticles (∼80 nm length and ∼20 nm width) caused mitochondrial energy metabolism failure in liver tissues. Following the buildup of nanoparticles in hepatic tissues, the scientists observed significant oxidative stress and impairment of mitochondrial respiratory enzyme performance. Additional *in vivo* investigation showed that oxidative damage caused by nanoparticles was linked to altered ATP generation and malfunction of the mitochondrial membrane. These results indicated that, especially at high doses and lengthy exposure times, prolonged nanoparticle retention in liver tissues may be a factor in metabolic toxicity and organ stress.^99^ These results show that although nHAp has intriguing biological potential, long-term organ buildup and excessive exposure may cause oxidative stress, mitochondrial impairment, and vascular dysfunction *in vivo*. To guarantee safe clinical translation, it is crucial to carefully optimize nanoparticle concentration, shape, and exposure circumstances.

The information compiled in Table 5 shows that particle physicochemical properties, such as size, morphology, crystallinity, aggregation behaviour, concentration, and surface modification, have a significant impact on the toxicity behaviour of nHAp composites. Higher exposure levels and extended retention may cause oxidative stress, inflammation, mitochondrial dysfunction, genotoxicity, and organ-related toxicity, even though lower concentrations of nHAp often show favourable cytocompatibility and biological performance. The table also emphasizes how dopants and surface engineering techniques have a major impact on cellular behaviour, immunological response, and ROS production. Overall, our results highlight how crucial it is to carefully optimize exposure settings and nanoparticle design in order to strike a compromise between the long-term biosafety of nHAp-based biomedical composites and their therapeutic effectiveness.

**Table 5:** An Overview of toxicity aspects, experimental observations and factors affecting the toxicity of nHAp

Toxicity aspect	Key findings	Factors affecting toxicity	Major observation on toxicity	References
Cell viability	Cell viability generally remained >80–90% at nHAp concentrations below ∼50–100 µg mL^−1^	Particle concentration, exposure duration, particle size	Lower concentrations exhibited favourable cytocompatibility in osteoblast and fibroblast cells	78 and 79
High-dose cytotoxicity	Exposure above ∼100–200 µg mL^−1^ significantly reduced proliferation and metabolic activity	High dose, prolonged exposure, aggregation	Toxicity strongly depended on exposure duration, particle size, and morphology	14, 24 and 80
Size-dependent toxicity	∼80 nm HAp particles induced greater apoptosis and reduced osteoblast proliferation compared to smaller particles	Particle size, cellular internalization	Particle size strongly affected cellular uptake and biological response	35
Shape-dependent toxicity	Rod/needle-like particles caused higher cytotoxicity than spherical particles	Morphology, surface roughness, aspect ratio	Surface topography and morphology influenced osteoblast adhesion and differentiation	35 and 83
Crystallinity effect	Low crystallinity promoted improved cell adhesion and differentiation	Crystallinity, surface chemistry	Crystallinity and surface chemistry regulated osteogenic behaviour	81, 82 and 84–86
Ag-doped nHAp toxicity	Ag concentration of 1.00 mol% showed the highest antibacterial activity; lower Ag content (0.5 mol%) showed selective antibacterial behaviour with improved biosafety	Ag^+^ ion release, dopant concentration, nanoparticle aggregation	Excessive Ag^+^ release and nanoparticle aggregation reduced mammalian cell viability	69
Optimized Ag-doped composite	Agx/HAp prepared by reverse precipitation with *x* = 0.2–0.3 showed the best balance between antibacterial activity and biocompatibility	AgNP dispersion, incorporation method, surface engineering	Homogeneous AgNP dispersion reduced aggregation-induced cytotoxicity	61
ROS generation	Colloidally stable nHAp showed ∼1.3-fold higher ROS signal than control; agglomerated nHAp showed ∼1.5-fold higher ROS signal than control	Aggregation, colloidal stability, crystallinity	Aggregation and colloidal instability enhanced oxidative stress behaviour	88
Surface-modified nHAp	Polydopamine-coated nHAp enhanced intracellular ROS and Ca^2+^ accumulation, activating JNK-mediated apoptosis	Surface coating, intracellular ion accumulation	Surface engineering significantly altered oxidative behaviour	90
Genotoxicity frequency	Out of 71 genotoxicity assessments, 20 studies showed positive genotoxic effects	Particle morphology, concentration, exposure time	Genotoxicity strongly depended on nanoparticle characteristics and exposure conditions	27
Immunotoxicity response	Micron-sized HAp induced stronger macrophage glycolytic activation than nanoHA	Particle size, macrophage uptake	Particle uptake altered macrophage metabolism and inflammatory signalling	96
*In vivo* vascular toxicity	Topical exposure of ∼1 g kg^−1^ nHAp significantly impaired microcirculation and altered vascular behaviour	Exposure route, local nanoparticle concentration	Toxicity strongly depended on exposure route and local concentration	15
Organ accumulation	nHAp (∼80 nm length, ∼20 nm width) accumulated in liver tissues and impaired mitochondrial respiratory enzymes	Biodistribution, particle retention, particle size	Long-term retention induced oxidative stress and ATP dysfunction	99

Although available animal studies indicate possible organ accumulation and vascular alterations following high-dose exposure, clinically relevant long-term toxicity data remain scarce. Most *in vivo* investigations evaluate acute responses using relatively high experimental doses that may not accurately represent therapeutic exposure. Future studies should focus on chronic biodistribution, biodegradation, clearance pathways, repeated-dose toxicity, and standardized Organisation for Economic Co-operation and Development (OECD)-guided *in vivo* protocols to facilitate regulatory approval and clinical translation of nHAp-based biomaterials.

Beyond publications, the nHAp market has grown with a number of patents and products for biomedical applications.^100^ The excellent bioactivity and osteoconductivity of nHAp make it an attractive material for bone tissue engineering, dental applications, implant coatings, drug delivery platforms, *etc*.^101^ Despite these significant advancements, the toxicity concerns of nHAp are still restricted for patients as well as commercialization products. Most of the patents focused on biological capacity rather than long-term safety and standard toxicity evaluation. This incongruity represents one of the major barriers preventing successful clinical translation of multifunctional nHAp composites. For instance, one patent discussed nano HAp coating with ZnO nanoparticles for orthopaedic and dental applications.^102^ Another patent claimed HAp as oral care compositions, which include reduced erosion of enamel with enamel repairing using these compositions.^101^ Although both technologies demonstrate improved biological performance, neither patent systematically establishes optimized nanoparticle concentrations, long-term degradation behaviour, nanoparticle release kinetics and chronic toxicity thresholds under physiological conditions.

The commercial translation of nHAp has been most advanced in dentistry, where nanohydroxyapatite has been added to fluoride-free toothpaste, remineralizing gels, oral rinses and desensitizing formulations. A recent patent application from the United States (20240148620) discloses a composition for remineralizing enamel and preventing caries that includes between 0.5 and 50 wt% nanohydroxyapatite (20–100 nm).^103^ Similarly, a number of oral-care products are available that take advantage of the nHAp's affinity for enamel to repair early carious lesions and to decrease dentinal hypersensitivity. However, commercial specifications tend to focus on the remineralization efficiency and whitening effect, while detailed data on chronic oral exposure, ingestion of nanoparticles, systemic distribution, and long-term safety are less abundant. Thus, product optimization has advanced more quickly than standardized toxicity validation. In addition, few patents incorporate internationally accepted safety assessment guidelines, such as OECD (Organisation for Economic Co-operation and Development) nanomaterial testing guidelines or ISO 10993 biocompatibility testing, in early-stage material development. The understanding of the combined effects of the particle composition, ion release, degradation rate, and cumulative exposure is even more critical for regulatory approval and clinical acceptance, as multifunctional nHAp composites are becoming more complex by incorporating reinforcing agents.

In general, the current patent and commercialisation situation shows that the technology innovation has progressed much more rapidly than the standardization of toxicity. Future studies should focus on the creation of safe designs of materials, where the evaluation of toxicity is considered from the initial design of the material. The use of standardized dose optimization, reproducible toxicological protocols and regulatory safety frameworks will significantly increase the translational consistency of nHAp-based products. This would not only speed up commercialization but also aid the creation of clinically viable biomaterials with predictable therapeutic performance that are safe for long-term use by patients.

Table 6 provides an overview of the 3P analysis, which includes publication outcomes, patent design focus and products related to nHAp composites. This critical comparison highlights that although research on nHAp has expanded significantly in terms of papers, patents and products, there is still a gap between available research and safety standards of nHAp composites.

**Table 6:** Publications, patents and product-based (3P) analysis of nHAp composites

3P component	Current status of nHAp technology	Highlights of the research	Toxicity-related gap
Publication Search	Extensive research on nHAp composites (>thousands of publications) covering bone regeneration, drug delivery, implant coatings, antimicrobial systems and imaging	Improvement of bioactivity, mechanical properties, antibacterial behaviour and therapeutic performance	Most studies focus on short-term *in vitro* biocompatibility; limited information on dose optimization, long-term exposure, biodistribution and chronic toxicity
Patent Search	Patents mainly target nHAp-based coatings, drug delivery implants, polymer-nHAp scaffolds and ion-substituted nHAp systems	Translation of nHAp composites into implantable materials, controlled release systems and regenerative platforms	Patent claims generally emphasize functional performance, while systematic toxicity validation, safe concentration limits and degradation-associated risks are rarely addressed
Product Search	Commercial nHAp products are available mainly in dental care (remineralizing toothpaste), bone graft substitutes and implant-related biomaterials	Consumer healthcare applications and clinical use of nHAp-based materials	Long-term repeated exposure, nanoparticle ingestion, systemic accumulation and regulatory-defined safety thresholds require further investigation

## 4. Toxicity aspects of nHAp in biomedical applications

**Fig. 8:**
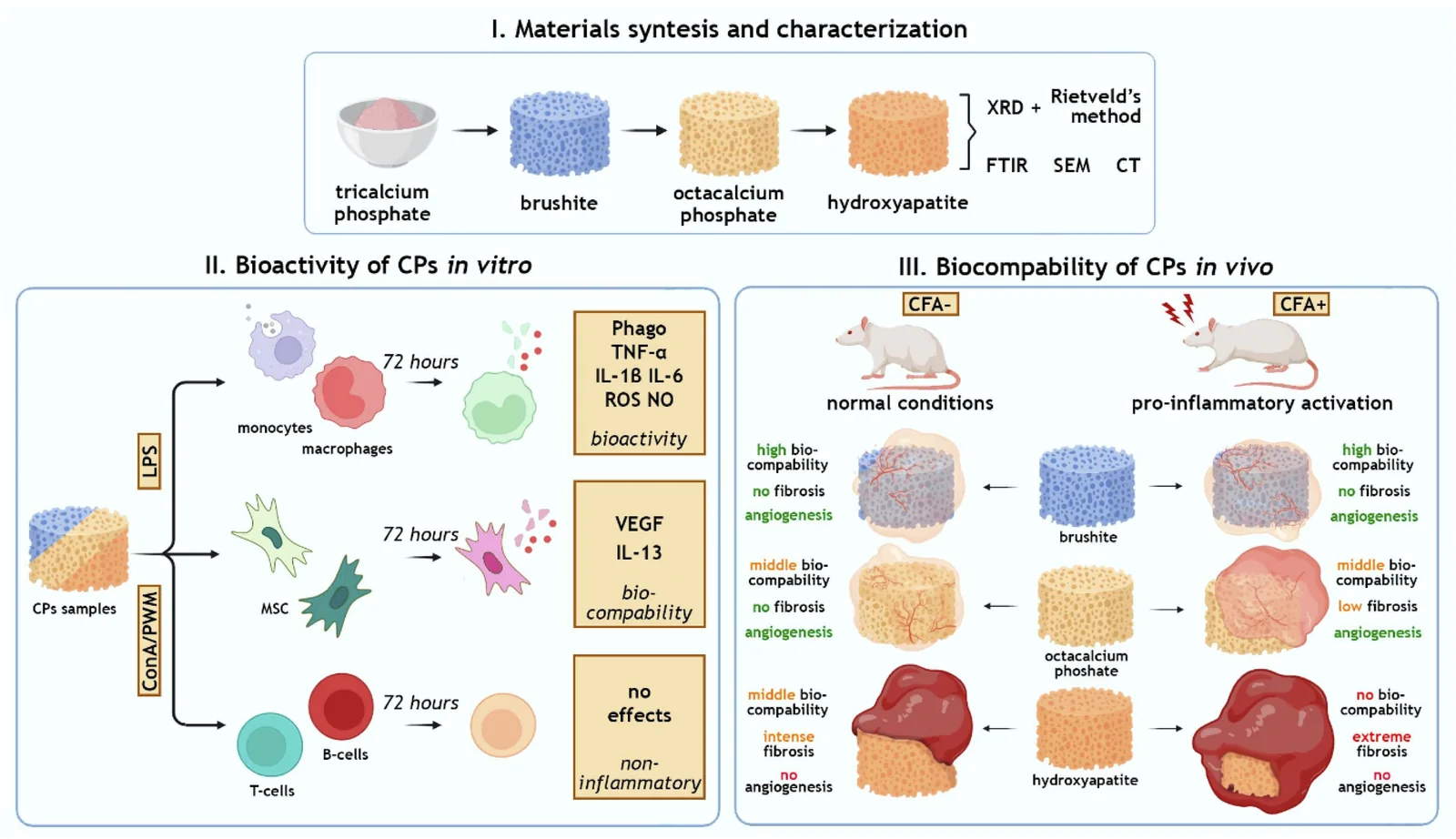
A schematic illustration of the synthesis and design of various calcium phosphate materials with their *in vitro* and *in vivo* studies under normal and inflammatory conditions. Reproduced with permission from ref. 106. Copyright 2026 MDPI (Open Access).

Numerous factors, such as severe traumas, accidents, infections, pathological diseases, and genetic illnesses, may result in bone deformities. As a result, effective and well-designed bone regeneration technologies are desperately needed. This objective has been made easier by developments in therapeutic approaches, with nHAp emerging as a widely researched and promising strategy in the area of bone tissue engineering.^104^ Hammal *et al.*^105^ designed a triple pod nHAp composite encapsulated with Mg, Si and Zn for bone regeneration. The composite showed refined crystallite size (∼17 nm) with enhanced surface roughness (*R*_a_ ∼6.8 nm), which led to improved performance of the nHAp. The significant biocompatibility was observed (>95% cell viability) with MG-63 cells and effective antimicrobial activity against *S. aureus* and *E. coli.* The authors concluded that the synergistic effect of dopants resulted in enhanced bioactivity. *In vivo* studies are crucial for a critical analysis of the biocompatibility of nHAp composites for translational applications. A recent study based on the *in vivo* investigations of nHAp composites exhibited that the biological activity of the HAp is more dependent on the host immune system than on the material.^106^ nHAp displayed significant biocompatibility in a rat model, but the testing on rats with Complete Freund's Adjuvant (CFA)-induced inflammation, nHAp triggered strong inflammation. This inflammation caused tissue necrosis, thick fibrous capsule formation, and graft rejection (Fig. 8). Thus, these results determined that *in vivo* safety and regenerative applications of nHAp are majorly reliant on the inflammatory microenvironment. Therefore, future preclinical assessment of nHAp-based composites should include disease-relevant inflammatory models to better envisage long-term clinical outcomes and ensure safe translation for bone regeneration applications.

A study by Liu *et al.*^107^ developed a customized structure from macro to nano structures of nHAp by a 3D printing process for bone tissue engineering. These composites displayed an improved protein adsorption, wettability, cell adhesion and proliferation, *in vitro*. Furthermore, the highest bone regeneration was observed (bone volume/total volume 42.8% and bone mineral densityv51.4 mg cm^−3^) in a rabbit defect model. Histological analysis further confirmed enhanced collagen deposition with significant osteointegration. No significant chronic inflammation was observed during *in vivo* studies, demonstrating amended biocompatibility. However, the scaffold exhibited slow degradation kinetics, indicating that long-term studies are still required to evaluate complete scaffold resorption and clinical translation.

Recently, Ardeshiriansharifabadi *et al.*^108^ fabricated nHAp with polycaprolactone (PCL) and coated it with zein for controlled release of tetracycline hydrochloride (TCH) in bone repair applications. The optimized scaffold contained 20 wt% of nHAp and 10 wt% TCH toward MG-63 osteoblast-like cells. Besides significant cytocompatibility, this scaffold showed improved antimicrobial activity against *E. coli* and *S. aureus.* According to the research, although high antibiotic loading may possibly hinder osteoblast proliferation due to localized toxicity effects, moderate TCH loading maintained adequate cell viability and encouraged cell adhesion and spreading. Furthermore, the controlled release behaviour decreased the abrupt exposure to surrounding tissues to high antibiotic concentrations, which is crucial for reducing cellular stress and inflammatory response during long-term implantation. These results show that drug concentration, release kinetics, coating homogeneity, and scaffold composition all significantly influence the toxicity profile of antibiotic-loaded nHAp scaffolds.

Dental caries and periodontal disorders, which affect both the teeth and the surrounding gingival tissues, are two of the most common oral health illnesses that people may experience throughout their lifetime.^109^ The remarkable properties of biomimetic nHAp, such as their biocompatibility, ability to alleviate inflammation, and lack of hazardous behaviour, allow for the remineralization of enamel surfaces.^110,111^ In preventative dentistry, nHAp have shown great promise, especially in the treatment of dentinal hypersensitivity, tooth erosion, and the remineralization of early carious lesions,^112^ but the optimized concentration is essential to maintain the non-toxicity of these nanoparticles. Hammal and co-workers^113^ developed nHAp loaded with clove extract (CE) for dental applications, jointly for remineralization and antibacterial ability. The size of nHAp was ∼80 nm, and the eugenol constituted approximately 72.5% of the clove extract composition, which exhibited good activity against oral germs. Thus, from the toxicity aspect, the particular size of nHAp, along with optimized concentration of essential oils such as eugenol are mandatory to avoid irritation, oxidative stress or cytotoxicity.

Medical imaging is a collection of techniques for photographing various body areas to identify health problems and monitor therapy progress. Creating novel imaging methods, including computed tomography, radiography, luminescence imaging, magnetic resonance imaging (MRI), and ultrasound.^114^ Traditional fluorescent dyes and fluorescent nanoparticles, which mainly consist of quantum dots, silica-based particles, and latex microspheres, are the two main types of probes used in optical bioimaging.^115^ By addressing the drawbacks of conventional fluorescent dyes, such as autofluorescence and poor photostability, as well as the toxicity, clearance, and solubility issues frequently seen with conventional fluorescent nanoparticles, nHAp offers a promising substitute for bioimaging applications.^114,116^ Recently, a multifunctional holmium-doped zinc oxide/nanohydroxyapatite nanocomposite functionalized with carbon dots (Ho^3+^:ZnO-HAp@CDs) was created for dual-modal imaging applications that combine fluorescence and magnetic resonance imaging (MRI).^117^ Due to the addition of 1.70 wt% Ho^3+^ ions, the nanocomposite displayed an asymmetrical shape with particle sizes in the range of around 110–120 nm and strong magnetic behaviour with a saturation magnetization value of 9.2607 emu/g. Crucially, the MTT test for toxicity assessment on L929 fibroblast cells showed that the nanocomposite was non-toxic up to a dose of 100 µg mL^−1^ after 24 hours of exposure, preserving adequate cell viability and morphology. These results imply that the multifunctional nHAp composite may provide imaging capabilities at modest doses without causing appreciable cytotoxicity. However, because ZnO-containing nanocomposites are known to produce ROS and release Zn^2+^ ions under physiological conditions, which may impact mitochondrial function and membrane integrity at elevated doses, the study also suggests that higher concentrations or extended exposure may potentially increase cellular stress. Additionally, the MCF-7 cancer cells' effective absorption of the nanocomposite demonstrates its potent cellular internalization capacity, which is beneficial for imaging but may potentially increase intracellular accumulation-related toxicity if the dose is not properly regulated. The findings show that dopant concentration, exposure time, cellular uptake, and metal ion release characteristics all significantly influence the toxicity behaviour of multifunctional imaging-oriented nHAp composites. Thus, the practical translation of luminomagnetic nHAp-based nanoprobes in biomedical imaging applications requires adjustment of particle composition and safe concentration limits.

Every year, millions of people are impacted by cancer, which is a major worldwide health concern. The use of nHAp-based technologies in cancer detection and treatment is receiving more attention. These strategies include phototherapy, targeted medication and gene delivery, sophisticated imaging technologies, and supplemental therapies to improve radiation therapy's effectiveness.^110^ Because of their strong ability to load and distribute drugs effectively, nHAp may be employed for cancer treatment in contrast to standard cancer theranostics.^118^ Additionally, magnetic nHAp are useful for delivering genes or plasmids and have a wide range of applications in bioimaging methods, which advances therapeutic and diagnostic modalities.^119^ Targeted delivery of anticancer medications to reduce off-target effects is a unique use of nHAp.^120^ Bulbul *et al.*^121^ synthesized poly(ε-caprolactone)/nanohydroxyapatite/curcumin (PCL/nHAp-Cur) fibrous mats for breast cancer therapy and confirmed that toxicity behaviour was strongly reliant on nHAp concentration. Various concentration of nHAp was tested (1, 3, and 5 wt%), and it was demonstrated that among all concentrations, 3 wt% was optimized formulation for controlled drug release and improved cancer treatment. This concentration reduced MDA-MB-231 breast cancer cell viability to approximately 45% after 48 h exposure, while maintaining high viability (>87%) toward normal L929 fibroblast cells. On the other hand, a higher nHAp loading (5 wt%) triggered a rapid burst release, with increasing curcumin release reaching ∼80% within 24 h, which may potentially increase localized cytotoxic stress and reduce long-term release control. According to the study, excessive nHAp incorporation changed fiber shape and drug diffusion behaviour, which may lead to unregulated drug exposure and potentially harmful cellular consequences under long-term exposure settings.

Overall, the studies discussed in this section demonstrate that the toxicity behaviour of nHAp composites in biomedical applications is highly application-specific and strongly influenced by composition, nanoparticle loading, release kinetics, degradation behaviour, and exposure conditions. Carefully optimized formulations generally exhibit favourable biocompatibility and therapeutic performance, whereas excessive nanoparticle concentration, uncontrolled burst release, rapid degradation, or high metal ion release may induce oxidative stress, inflammatory response, and cellular toxicity. Crucially, a number of studies have shown that intermediate nanoparticle concentrations often provide the optimal compromise between biosafety and biological effectiveness. These results highlight the need for precise control over drug release behaviour, long-term biological interactions, and nanoparticle engineering for the effective clinical translation of nHAp-based systems. Future biomedical designs should thus prioritize lowering toxicity and guaranteeing long-term biosafety under clinically relevant settings in addition to enhancing therapeutic usefulness.

## 5. Research gap, translational challenges and future prospects

Despite the noteworthy advances in the field of nHAp composites, their translational growth is still challenging due to the unexplored scientific and regulatory limitations. Most researchers have focused on improving mechanical, tribological, biocompatibility, antimicrobial activity, and drug delivery efficiency; however, a systematic study on toxicity, bio-safety and application-specific safety remains scarce in the literature.^4,122,123^ Thus, a thorough investigation into the toxicity and data optimization of nHAp composites can offer potential guidance for researchers for the fabrication of safe and effective composites for translational applications.^61,74^ The critical analysis of nanoparticle morphology and *in vivo* studies is also lacking in the literature, which limits the translation application and provides a major research gap in this field. A recent study on genotoxicity showed that needle-shaped and rod-shaped nanoparticles exhibited higher toxicity by damaging DNA than spherical nanoparticles. The study highlighted that very limited studies are presented in the literature, which reported *in vivo* safety evaluation.^27^

Moreover, international safety frameworks published by the organization for economic co-operation and development (OECD) suggested tiered testing strategies involving physicochemical characterizations, computational prediction, *in vitro* and carefully designed *in vivo* studies for nanosafety predictions. A major challenge, which restricts the translational applicability of nHAp composites, is the absence of standard dose recommendations for various biomedical applications. The nHAp dose can be considerably changed as per the applicability, ranging from implant coatings to dental, drug delivery, cancer therapy or imaging.^124,125^ Encouragingly, recent regulatory evaluation by the Scientific Committee on Consumer Safety (SCCS, European Commission) concluded that nano-hydroxyapatite is safe for oral-care products under defined particle specifications and concentration limits (up to 29.5% in toothpaste and 10% in mouthwash), illustrating the importance of defining application-specific safety windows rather than assuming universal biocompatibility.

While significant advances have been made in the knowledge of the toxicological behaviour of nHAp composites, there are still some limitations. The majority of the studies currently available are short-term *in vitro* studies, and there is a lack of studies to address long-term *in vivo* toxicity, chronic exposure behaviour, biodistribution, metabolic fate and clearance mechanisms. Moreover, there are substantial differences between the synthesis procedures, the shape of the particles, the experimental design and the biological models used in different studies. Another difficulty is the absence of standardized toxicity evaluation protocols to establish definitive safety thresholds for clinical applications. Standardized and reproducible toxicological assessment frameworks for nHAp-based systems should thus be developed for future research. Long-term biodistribution, immunological interactions, vascular toxicity and degradation behaviour under clinically relevant conditions are key areas of study that urgently require investigation. Surface engineering, green synthesis methods, and smart multifunctional coatings with reduced oxidative stress and inflammatory response, but maintaining therapeutic activity, should also be focused on. Moreover, the use of artificial intelligence for material optimization, patient-specific biomaterials, and stimuli-responsive nHAp composites could be significant future trends in the field of safer and more efficient biomedical applications.

The use of computational modelling in conjunction with experimental toxicology is likely to be an important pathway for the safe design of nHAp-based biomaterials. While *in vitro* and *in vivo* studies have been used in most toxicity investigations, computational methods are relatively less explored for predicting the biological activity of nHAp composites. Atomic-level understanding of dopant incorporation, surface reactivity, electronic structure, and ion-release behaviour can be obtained using density functional theory (DFT), and molecular dynamics (MD) simulations can explain the interactions between nanoparticles and cell membranes, proteins, and biomolecules in physiological conditions. These methods could be useful to elucidate the mechanism of oxidative stress, internalization and protein corona formation prior to broad biological testing. The recent developments in artificial intelligence (AI) and machine learning (ML) also offer new possibilities to develop quantitative structure–toxicity relationships by combining experimental data with material descriptors like particle size, morphology, crystallinity, surface charge, concentration, and exposure time.^126,127^ But computational studies on the toxicity of nHAp are still scarce, and most of the models available are mainly based on structural optimization or mechanical properties and not on nanosafety assessment.^128,129^ Future research should thus integrate DFT, MD simulations and AI/ML-driven predictive modelling with standardized experimental validation to determine safe concentration ranges, optimize composite composition and expedite rational development of clinically translatable and biosafe nHAp-based materials.

## 6. Conclusions and outlook

The composites based on nHAp have proven to be very promising biomaterials for various biomedical applications due to their excellent bioactivity, osteoconductivity, biomimetic nature and multifunctionality. Their application potential has been further extended by incorporating reinforcing agents like polymers, metals, ceramics and carbonaceous materials in bone tissue engineering, implant coatings, drug delivery, antimicrobial systems and regenerative medicine. These hybrid composite systems have superior mechanical strength, enhanced biological interactions, controlled drug release behaviour, antimicrobial activity and superior tissue integration when compared to conventional hydroxyapatite systems.

However, the present review shows that the toxicity of nHAp composites is very complex, multifactorial and is strongly dependent on physicochemical properties and exposure conditions. Their biological response is critically dependent on their parameters, such as particle size, morphology, crystallinity, concentration, aggregation behaviour, surface charge, dopant composition, degradation kinetics, and exposure duration. Lower concentrations and well-dispersed spherical nanoparticles typically have good cytocompatibility and minimal toxicity, but higher concentrations, longer exposure times, and rod or needle-shaped particles can lead to oxidative stress, mitochondrial dysfunction, inflammatory signalling, DNA damage, macrophage activation, and organ-specific toxicity. Additionally, the addition of metallic dopants like Ag, Cu, and Zn can greatly improve the antibacterial properties but can also cause high cytotoxicity due to high release of ions and formation of ROS, if not properly controlled.

The review also points out that toxicity behaviour is application-specific. Long-term degradation and persistence of nanoparticles can affect tissue remodelling and inflammatory response in bone tissue engineering. Burst release and accumulation of carrier are still significant issues in drug delivery systems, while coating wear under physiological loading conditions can produce nanoscale wear debris. Likewise, nHAp systems with antimicrobial properties must be optimized for both antimicrobial activity and mammalian cell compatibility. Thus, optimization of engineering strategies for nanoparticles is crucial for safe clinical translation. In summary, nHAp composites have tremendous potential in the field of modern biomedical engineering; however, a balance of knowledge on the therapeutic and toxicological aspects of these composites is necessary for their successful clinical translation. Careful optimization of composition, morphology, dosage, and exposure conditions will be crucial for the development of safe, effective, and clinically reliable nHAp-based biomaterials in the future.

## Conflicts of interest

There are no conflicts to declare.

## Data Availability

No primary research results, software or code have been included and no new data were generated or analysed as part of this review.
